# Emotional Experiences of the Home‐Dwelling Older Adults During the Isolation of the Coronavirus Disease 2019 Pandemic: A Qualitative Systematic Review

**DOI:** 10.1002/hsr2.71614

**Published:** 2025-12-03

**Authors:** Zeinab Dolatshahi, Pouran Raeissi, Shahin Nargesi, Nadia Saniee

**Affiliations:** ^1^ Student Research Committee Iran University of Medical Sciences Tehran Iran; ^2^ Department of Healthcare Services Management School of Health Management and Information Sciences Iran University of Medical Sciences Tehran Iran; ^3^ Department of Health Management and Economics Faculty of Health Ilam University of Medical Sciences Ilam Iran; ^4^ Department of Basic Sciences Asadabad School of Medical Sciences Asadabad Iran

**Keywords:** aged, coping strategies, COVID‐19 pandemic, emotional resilience, isolation, older adults, qualitative systematic review

## Abstract

**Background and Aims:**

This systematic qualitative review aimed to provide an in‐depth understanding of the emotional experiences and coping strategies of home‐dwelling older adults, as expressed in their own words, during the quarantine period of the COVID‐19 pandemic.

**Methods:**

Electronic searches were conducted in PsycINFO, CINAHL, Scopus, Web of Science, PubMed, and other related databases. Articles published between January 2020 and December 2021 were identified using predefined keywords. After screening studies based on inclusion and exclusion criteria, relevant data were extracted, and the results were synthesized. The quality of the included studies was assessed using the COREQ checklist. A total of 43 qualitative studies were included in the final review.

**Results:**

Based on COREQ evaluation, most studies demonstrated good methodological quality, with a mean score of 25.81 out of 32 (range: 17–29); no study fully satisfied all COREQ criteria. Thematic synthesis revealed two main categories: (i) Emotional Challenges, which encompassed psychological, physical, technological, and social dimensions; (ii) Coping Strategies, which were classified into cognitive (mindset‐based), behavioral (function‐based), and technology‐assisted strategies. These findings highlight the emotional complexity and adaptability of older adults during isolation.

**Conclusions:**

Contrary to initial assumptions of vulnerability, many older adults approached the pandemic with rational understanding and adaptive responses. By drawing on past life experiences, they actively organized coping mechanisms to navigate the crisis. These insights emphasize the need for health policymakers to invest in resilience‐building initiatives, such as digital literacy training and community‐based emotional and physical support programs. Such strategies can enhance the quality of life for older adults and promote efficient resource utilization within health systems.

## 1 Introduction

Severe Acute Respiratory Syndrome Coronavirus 2 (SARS‐CoV‐2), the causative agent of Coronavirus Disease 2019 (COVID‐19), emerged in Wuhan, China in December 2019 [1]. The swift global spread of the disease propelled the World Health Organization (WHO) to declare it a pandemic on March 11, 2020 [2, 3]. The impact of the COVID‐19 pandemic on older adults (individuals aged 60 or older) has been so profound that researchers have described it as a “geriatric health emergency” [4, 5]. Scientific evidence indicates that the immune systems of older adults individuals are less resistant to diseases and infections, making exposure to COVID‐19 particularly detrimental, leading to increased physical and psychological damage and prolonged recovery times [6, 7].

Global mortality data underscore this vulnerability: As of April 30, 2021, global reports from 210 countries revealed ~150 million infected cases and more than 3.2 million deaths [8, 9]. The CDC confirmed that older adults (65 years and over) demonstrated higher risk of developing severe COVID‐19 compared to younger populations, with no significant gender‐related differences in disease severity [10]. China's Center for Disease Control and Prevention reported that the death rate for individuals aged 60–69 years was 3.6%, escalating to 18% for those aged 80 and over [11].

Following WHO's pandemic declaration, governments worldwide implemented infection control measures including enhanced hygiene protocols, social distancing, and various forms of isolation and movement restrictions [12].

According to Kermes and colleagues, loneliness occurs when social relationship networks, either quantitatively or qualitatively, fail to meet individual needs, creating an unpleasant subjective experience. Isolation is defined as the objective absence of reciprocal social interaction [13]. “Social distancing” encompasses the deliberate maintenance of physical distance to prevent disease transmission, while “quarantine” specifically refers to the restriction of movement for individuals who may have been exposed to the virus [14].

While these public health interventions were designed to protect older populations, they inadvertently created negative effects on the health and welfare of this vulnerable group [15]. This social connectivity paradox demonstrates that while social distancing and isolation measures reduced COVID‐19 exposure, they simultaneously increased loneliness and social isolation [16].

Self‐assessment plays a crucial role in older adults individuals' general health and mortality risk, with those experiencing depression or emotional difficulties demonstrating pessimistic health self‐assessments and higher mortality risk, while those maintaining positive life experiences generally exhibit better overall health [17, 18]. “Emotional experiences” in this context encompass the full spectrum of psychological responses, including but not limited to anxiety, depression, fear, resilience, and adaptive coping mechanisms [19].

Previous research has extensively documented the general health impacts of COVID‐19 on older adults, yet significant gaps remain in our understanding of their lived experiences during pandemic restrictions. While quantitative studies have measured rates of depression, anxiety, and isolation among older adults populations during COVID‐19, there is limited qualitative exploration of how older adults personally experienced and coped with these challenges.

Recent studies have highlighted the importance of family structures and social support systems for older adults' well‐being. Research by Prabhakar and colleagues explored forgiveness patterns among Indian older adults in different living arrangements, revealing significant differences between those in institutional care versus family settings [20]. Similarly, Tiwari and colleagues demonstrated that forgiveness attributes and family relationships play crucial roles in later life adaptation [21]. Furthermore, Tiwari and colleagues showed that family structures (joint vs. nuclear families) provided protective roles for children during COVID‐19 lockdowns, suggesting similar protective mechanisms may operate for older adults [22].

Despite extensive research on the impact of the COVID‐19 pandemic on older adults, their lived experiences and personal interpretations during pandemic‐related restrictions have received limited attention. This study adopts a qualitative approach to address this study gap by exploring how older adults experienced, made sense of, and coped with isolation, and by examining the role of media and online technologies in their adaptation process.

The findings of this study can inform evidence‐based interventions aimed at enhancing the mental health and resilience of older adults in similar crises, and—alongside existing quantitative data—offer a more comprehensive understanding of how this population navigates public health emergencies.

## 2 Materials and Methods

This review seeks to provide a configurative and interpretive synthesis of qualitative studies that explored the emotional experiences and coping strategies of home‐dwelling older adults individuals during the COVID‐19 quarantine period. To achieve this objective, the following research questions were addressed:

What were the emotional experiences of older adults people during the COVID‐19 quarantine worldwide?

What coping strategies did they adopt during this period?

A qualitative meta‐ethnography approach was employed to conduct this synthesis. This method was selected over other review methodologies because it enables researchers to go beyond the mere aggregation of data and instead generate new conceptual understandings by interpreting and translating key themes and metaphors across multiple studies. Given the nature of our research questions—which focus on subjective experiences and coping mechanisms—meta‐ethnography provided a rigorous and suitable framework for capturing the depth and complexity of qualitative data.

To ensure methodological transparency and adherence to best practices, this review followed the seven‐phase framework developed by Noblit and Hare, which includes: (1) Specifying the research question, (2) Selecting relevant studies, (3) Detailed study and data extraction, (4) Determining the relationships between studies, (5) Translating studies (interpreting), (6) Combining interpretations, and (7) Presenting the final report [23]. Although the review presents thematic descriptions, the meta‐ethnographic process enabled a deeper level of interpretation through the reciprocal and refutational translation of concepts, resulting in a line‐of‐argument synthesis that provides new insights into how older adults individuals emotionally navigated the pandemic and the strategies they used to maintain resilience [23, 24].

This review was conducted in accordance with the Preferred Reporting Items for Systematic Reviews and Meta‐Analyses (PRISMA) guidelines [25].

### 2.1 Literature Search Strategy

An electronic search strategy, including the terms “aged,” “geriatrics,” “COVID‐19 pandemic,” “SARS‐CoV‐2,” “mental health,” “older adults experience,” “resilience,” and “qualitative study” (along with their associated MeSH terms), was employed to identify the relevant evidence.

We primarily used PubMed as our main database because it provides free access to a wide range of biomedical topics. It covers various fields within medical sciences, especially nursing, dentistry, and veterinary medicine. Since this study relates to concepts in nursing and psychology, PubMed was the most suitable choice. We also included Web of Science and Scopus, as they are well‐known citation databases worldwide, covering multiple disciplines including medical sciences. These platforms help us track citations of relevant articles and discover other related studies. Additionally, we chose ProQuest because it encompasses a variety of subject areas, including medical sciences, and provides access to gray literature, which enhances the comprehensiveness of our literature search in systematic reviews.

Google Scholar was specifically searched using its “cited by” and “related articles” functions to broaden the scope of the retrieved studies.

Keywords were chosen based on prior literature, expert consensus, and their relevance to the main concepts of the study (i.e., aging, mental health, and qualitative research methodology). The inclusion of both general terms and controlled vocabulary (MeSH) ensured a sensitive and systematic search.

### 2.2 Selection of Studies

To maintain focus and avoid overgeneralization across diverse research fields, the scope of this meta‐ethnography was intentionally narrower than that of broader qualitative narrative reviews. Although such specificity may have led to the exclusion of certain studies, it allowed for a more manageable and in‐depth examination of the most relevant themes.

The analytical process involved systematically coding the findings of the included studies through repeated discussions within a multidisciplinary research team. This team included experts from psychology, health economics, health policy, and palliative care nursing. Such collaborative engagement enhanced both the credibility and validity of theme extraction and interpretation.

Phase 1 focused on synthesizing interview themes, older adults' attitudes, and the emotional experiences associated with pandemic‐related isolation. The aim was to identify the essential requirements for creating safe, supportive, and engaging environments for older adults in similar future crises. Ultimately, the synthesis sought to inform policymakers by offering robust, evidence‐based insights to guide efficient and effective interventions for ageing populations.

Phase 2 followed a structured and transparent study selection protocol consisting of four sequential steps:

1. Defining the focus of the synthesis to ensure alignment with the research question;
2. Establishing explicit and comprehensive inclusion and exclusion criteria;
3. Systematically identifying and selecting eligible studies through targeted database searches and supplementary sources; and
4. Conducting a rigorous methodological quality assessment of all included studies.

The study selection procedure was carried out as follows:

- Step 1: Duplicate records were removed from the initial pool of retrieved articles.
- Step 2: Titles and abstracts were screened for relevance according to the predefined eligibility criteria.
- Step 3: Full‐text versions of potentially eligible studies were retrieved and thoroughly evaluated against the comprehensive inclusion and exclusion criteria.
- Step 4: Final inclusion was limited to studies meeting all criteria after quality appraisal.

All screening and selection processes were performed independently by two reviewers. Any disagreements were resolved through discussion, and where consensus was not achieved, a third reviewer was consulted.

### 2.3 Inclusion Criteria

The inclusion criteria were developed based on the PICOTs framework, ensuring a structured approach to article selection. Studies were included if they met all of the following criteria:

i. Qualitative studies that explored the impact of the COVID‐19 pandemic on the mental health of home‐dwelling older adults individuals.
ii. Studies that investigated the emotional experiences of community‐dwelling older adults people resulting from quarantine or isolation due to COVID‐19.
iii. Studies that examined coping or resilience strategies adopted by the older adults during the pandemic‐induced quarantine.
iv. Studies that collected data through qualitative methods, including in‐depth interviews (face‐to‐face, telephone, or video calls) or focus groups.
v. Studies in which participants were over 60 years old, living in noninstitutional settings (i.e., in their own homes), and had no acute physical illnesses or diagnosed cognitive/memory impairments.

### 2.4 Exclusion Criteria

Studies were excluded if they met any of the following conditions:

i. Articles not written in English.
ii. Studies published outside the specified time frame (i.e., before January 2020 or after December 2021).
iii. Studies involving older adults participants living in institutional settings such as nursing homes or long‐term care facilities.
iv. Studies that did not employ primary qualitative data collection methods (e.g., case reports, reviews, conference abstracts, gray literature, editorials, or non‐peer‐reviewed materials).

### 2.5 Quality Assessment of the Entered Studies

The Consolidated Criteria for Reporting Qualitative Research (COREQ) checklist, consisting of 32 items across 3 domains, was used to assess the methodological quality of the included studies. Each article was independently evaluated by two reviewers who systematically assigned scores based on the degree of compliance with each item.

Items that were fully addressed received a score of “1” and were marked as “Y” (Yes). Items that were partially addressed were assigned a score of “0.5” and marked as “P” (Partial), while items not addressed at all were given a score of “0” and marked as “N” (No).

Discrepancies between reviewers were resolved through discussion with a third reviewer, ensuring consistency and objectivity in the assessment process. Using established criteria, COREQ compliance was coded either excellent (≥ 30 items), good (≥ 25 items), moderate (17–24), poor (9–16), very poor (≤ 8) based on the number of items addressed in each study [26].

This scoring system enabled a structured evaluation of the transparency and rigor of qualitative reporting, allowing us to differentiate studies based on the robustness of their methodological reporting.

### 2.6 COREQ Compliance

Table 1 presents the compliance rates of the included studies with the 32 individual items of the COREQ checklist. The mean score per study was 25.81 out of 32, with scores ranging from 17 to 29. None of the studies fully complied with all 32 criteria.

**Table 1:** Number and percentage of studies reporting against each COREQ item.

COREQ items	Reporting criteria/*n* (%)
*Domain 1: Research team and reflexivity*	
Personal characteristics	Average: 19 (44%)
1. Interviewer/facilitator identified	27 (63%)
2. Credentials (e.g., PhD)	30 (69%)
3. Occupation at the time of the study	22 (51%)
4. Gender of interviewer	7 (16%)
5. Researcher experience and training	11 (25%)
Relationship with participants	Average: 10 (23%)
6. Relationship established	14 (32.5%)
7. Did the participants know about the researcher	9 (21%)
8. Researcher characteristics	8 (18.6%)
*Domain 2: Study design*	
Theoretical framework	Average: 43 (100%)
9. Methodological orientation	43 (100%)
Participant selection	Average: 31 (72%)
10. Sampling	43 (100%)
11. Method participants approached	32 (74%)
12. Number of study participants	43 (100%)
13. Number of participants that refused to participate or dropped out	6 (24%)
Setting	Average: 30 (69%)
14. Setting of data collection	43 (100%)
15. Others present during interview	6 (13%)
16. Description of sample	40 (93%)
Data collection	Average: 29 (66%)
17. Use of topic guide	42 (99%)
18. Repeat interviews conducted	5 (12%)
19. Audio or visual recording	43 (100%)
20. Field notes	15 (35%)
21. Duration of the interviews or focus groups	41 (95%)
22. Data saturation	36 (84%)
23. Transcripts returned	18 (42%)
*Domain 3: Analysis and findings*	
Data analysis	Average: 19 (44%)
24. Number of data coders	20 (48%)
25. Description of the coding tree	15 (35%)
26. Themes identified in advance or derived from the data	35 (81%)
27. Software used	14 (32.5%)
28. Participant feedback	10 (23%)
Reporting	Average: 41 (95%)
29. Quotations used to illustrate themes/findings	38 (88%)
30. Consistency between data and findings	42 (98%)
31. Clarity of major themes	41 (95%)
32. Description of diverse cases or discussion of minor themes	42 (98%)
Supplementary items	Average: 26 (60%)
Study limitations reported	37 (86%)
Ethical issues reported	36 (84%)
Authors reported they followed COREQ	4 (9%)

Among the three COREQ domains, Domain 2 (Study Design) demonstrated the highest overall compliance, with an average score of 77% in over 80% of the studies. This domain includes categories such as the theoretical framework (addressed in 100% of the studies), participant selection (72%), and setting and data collection procedures (~70%).

Domain 3 (Analysis and Findings) ranked second, with 66% of the studies addressing its criteria. Notably, the reporting subdomain within this category showed the strongest performance, with a mean coverage of 95%, indicating that most studies clearly reported findings and provided supporting quotations or interpretations. Supplementary items and data analysis procedures were also generally well addressed in this domain.

In contrast, Domain 1 (Research Team and Reflexivity) received the lowest attention, with only 33.5% compliance. This domain includes items such as researcher personal characteristics and their relationship with participants, suggesting that many studies underreported potential sources of bias or reflexivity components.

Overall, based on COREQ criteria, the methodological quality of the included studies was rated as good in 67% of cases and moderate in 33%, reflecting a generally acceptable level of reporting rigor and transparency across the reviewed literature.

## 3 Results

### 3.1 Literature Search Result

**Figure 1:**
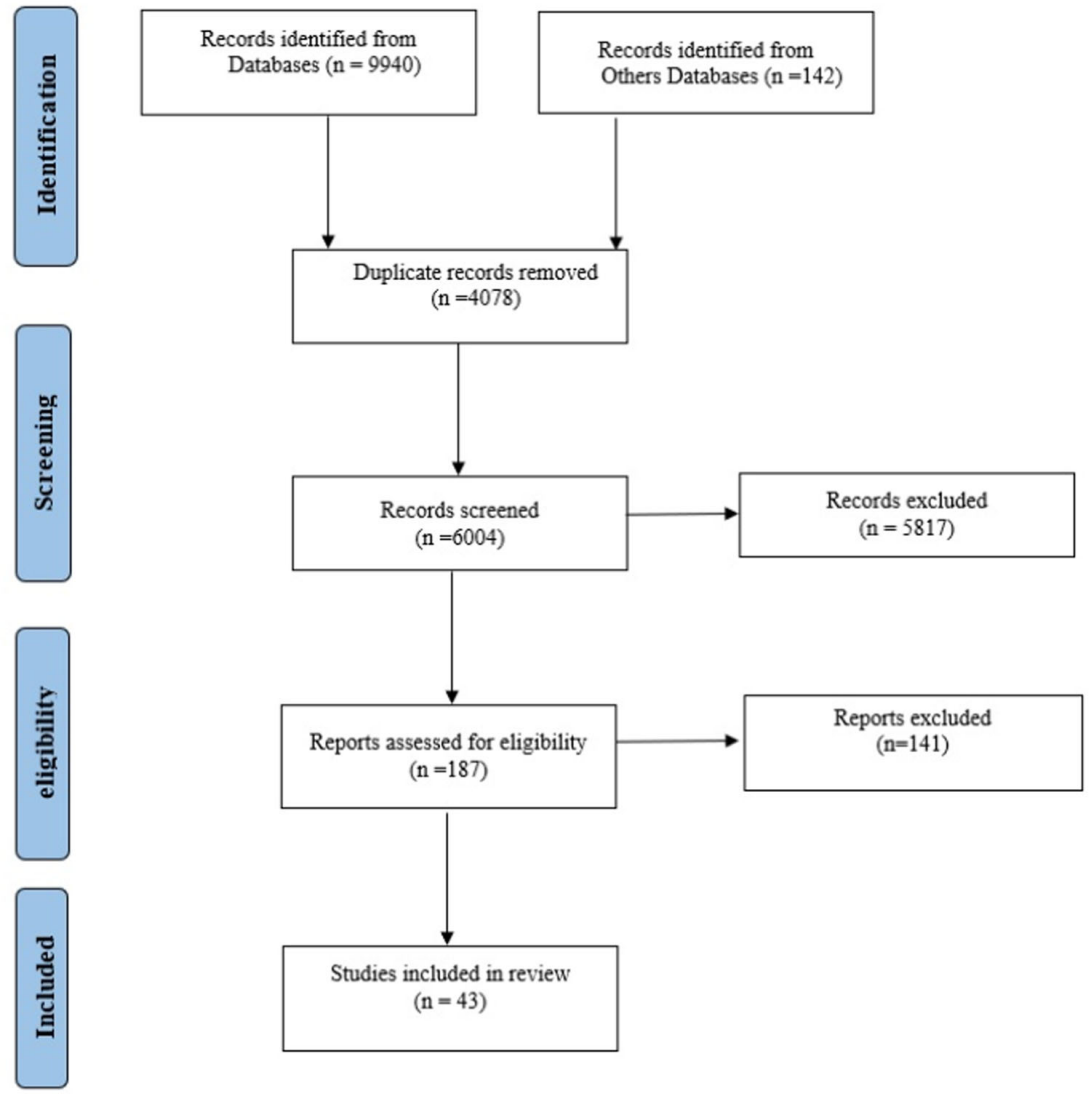
Process of the systematic literature search, according to the Preferred Reporting Items for Systematic Review.

The initial search retrieved 10,082 related citations, of which 1707 were from PubMed, 5030 from Web of Science, 2091 from Scopus, 1112 from ProQuest, 121 from Google Scholar, and 21 from other databases. After the removal of 4078 duplicate records, the remaining 6004 studies were screened by title and abstract. A total of 3897 studies that did not meet the inclusion criteria based on their abstracts were excluded. Full‐text screening was conducted for 2107 articles. Of these, 1051 articles were excluded due to inappropriate study design, 799 articles failed to meet adequate reporting or methodological standards as assessed by the COREQ checklist, and 27 articles were excluded due to lack of full‐text access. In total, 187 full‐text studies were assessed for eligibility, and 43 articles were finally included in the review. Figure 1 shows the study selection process according to PRISMA guidelines. Figure 1 shows the review selection strategy based on the PRISMA guidelines.

### 3.2 Characteristics of the Studies

In total, 43 of the selected studies published from January 2020 to the end of December 2021 were included in this review. Seven studies were from the United States [27, 28, 29, 30, 31, 32, 33], four from Canada [34, 35, 36, 37], three from the United Kingdom [38, 39, 40], three from India [11, 41, 42], two from Argentina [43, 44], two from Finland [45, 46], two from Hong Kong [47, 48], two from Iran [49, 50], two from Italy [51, 52], two from the Netherlands [53, 54], two from Spain [55, 56], and one from each of the following countries: Brazil, China, Ghana, Japan, Nigeria, Philippines, South Korea, Sweden, and Turkey [57, 58, 59, 60, 61, 62, 63, 64, 65]. Three of these studies originated from Ph.D. theses, part of an OTD (Doctor of Occupational Therapy) capstone project, and the Faculty of Spatial Sciences [66, 67, 68]. The selected studies focused on home‐ or community‐dwelling older adults populations with no physical strains and free of cognitive and memory deficits.

The included studies had varying numbers of participants. Two US studies had a maximum of 76 participants each [28, 32], and two studies from the United States and Philippines had a minimum of 5 participants each [62, 67]. The cumulative number of participants was 1023, and the average number of participants in all selected studies ranged between 23 and 76.

All of the studies were conducted as qualitative analyses; six studies used mixed methods [15, 28, 32, 40, 41, 48], six studies were conducted using the phenomenological method [38, 49, 50, 51, 53, 58], four employed the exploratory method [36, 43, 44, 56], one used the case study method [62], one applied the narrative method [42], and the remaining studies used the descriptive method.

Sampling methods varied: 12 studies used convenience sampling [27, 29, 31, 32, 38, 40, 55, 56, 57, 65, 67, 68], 2 used community‐based sampling [28, 35], 8 used snowball sampling [34, 41, 43, 44, 52, 53, 60, 63], 13 employed purposive sampling [11, 36, 39, 42, 45, 46, 47, 48, 49, 50, 54, 58, 59], 5 drew samples from previous primary studies such as clinical trials or longitudinal studies [30, 33, 37, 64, 66], 1 study used random sampling [61], and 1 combined convenience and snowball sampling [51].

Data collection methods included in‐depth interviews and focus groups in one study [41], semi‐structured interviews and focus groups in two studies [33, 56], a combination of semi‐structured in‐depth interviews in five studies and in‐depth plus unstructured interviews in one study [27, 42, 47, 48, 49, 58], purposive and snowball mixed methods in one study [62], and almost all remaining studies used semi‐structured or in‐depth interviews conducted via phone or video call.

For data analysis, Collazzi's method was used in two studies [50, 59], the seven steps of Diekelman's approach in one study [49], grounded theory in five studies [31, 55, 58, 65, 66], three studies used a conceptual framework [43, 44, 54], and the remaining studies applied conventional or content analysis or thematic analysis, either mixed or alone.

The mean age across studies was ~70 years, with more than 60% (64.5%, equal to 670 individuals) of participants being women. However, in four studies [60], men outnumbered women [41, 49, 50, 66], including one study with 100% male participants [49]. Most studies were conducted during the early weeks of the pandemic in their respective countries or cities. Main characteristics of the included studies are presented in Supporting Information S1: Table 2.

### 3.3 Main Explored Themes of the Studies

The qualitative data on the emotional experiences of the older adults from the included studies were categorized into several main themes, detailed in Supporting Information S1 and S2: Tables 2 and 3 (see Supporting Materials). To identify the most significant themes, the authors conducted regular coding sessions and discussions with the analytic team. In the first phase, the authors aimed to synthesize the themes from the interviews, focusing on the older adults's attitudes and emotional experiences during pandemic‐induced isolation. This synthesis was intended to help understand the needs necessary to create safe and comfortable conditions for older adults individuals in similar situations, based on their lived experiences and perceptions. We divided the process into the following stages: reciprocal synthesis and line of argument synthesis.

These stages allowed us to establish relationships between the studies' themes and to approach a reciprocal synthesis. During the reciprocal phase, we summarized the shared themes across the studies by juxtaposing first‐ and second‐order constructs. This led to the generation of new concepts that provide a fuller account of the phenomenon and resolve any contradictions; these are known as the original third‐order constructs developed by the review authors and provide a new understanding of the phenomena. At the line of argument phase, we created a synthesis from the third‐order constructs (known as higher‐order interpretations), achieved by continual comparison of concepts and elaborating a grounded theory that organizes similarities and differences between the studies interpretatively.

The final phase included the following three stages: (i) Summary of findings, (ii) Strengths, limitations, and reflexivity, and (iii) Recommendations and conclusions.

A broad range of populations and realistic emotional experiences were included in the review and are presented within the synthesis. The problem of lack of consensus between coders was resolved by the lead researcher (first author), acting as a third coder and reviewing the relevant text lines where themes were identified. Finally, the lead researcher reviewed all transcripts to identify exemplar quotes for each specific theme.

Although many older adults expressed positive perspectives and attitudes, not all participants shared this positive mindset. Some expressed both positive and negative emotions within the same interview. This variability is important because, despite many positive examples, some participants showed less positive or even negative mindsets.

**Figure 2:**
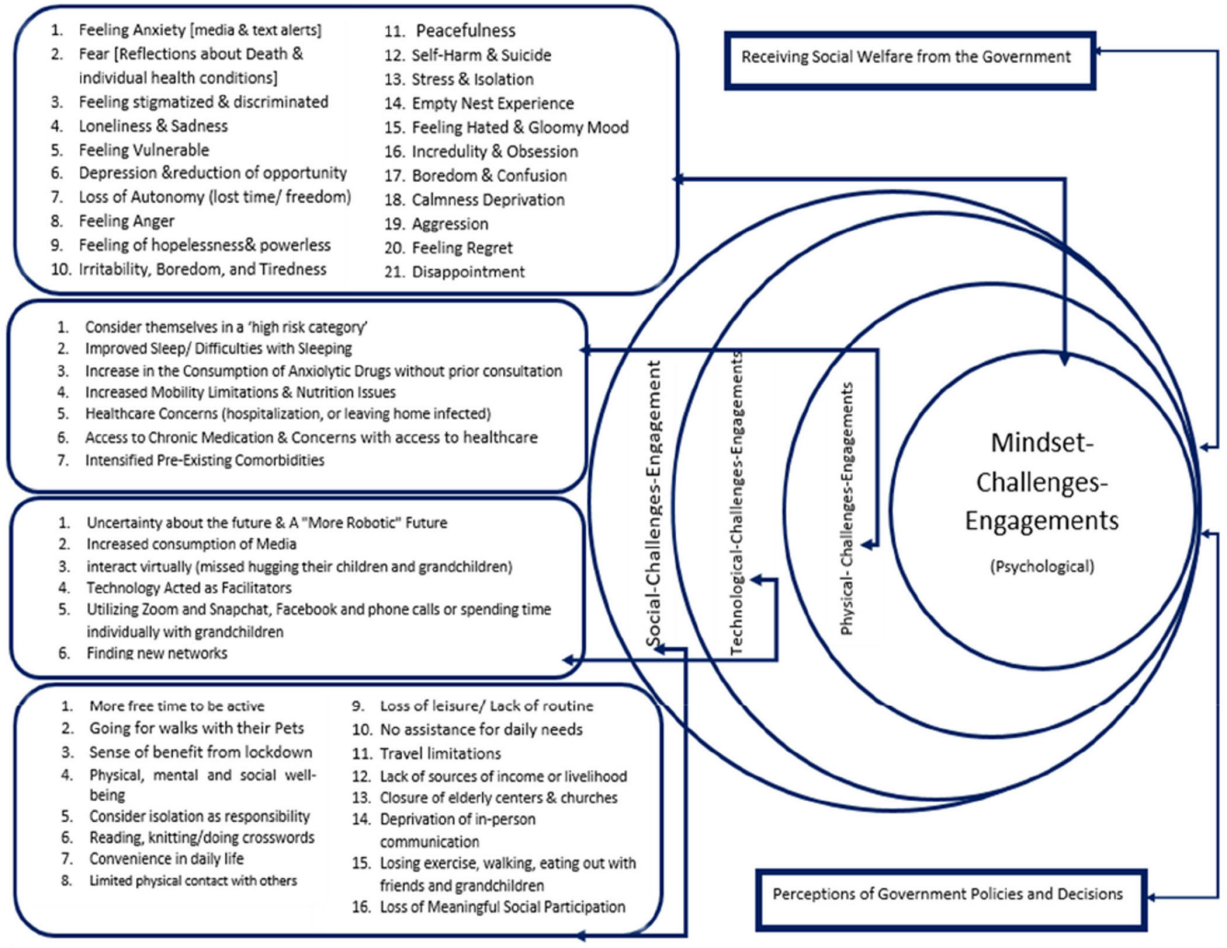
Main explored themes of the dimensions of the older adults experiences during the COVID‐19 pandemic.

**Figure 3:**
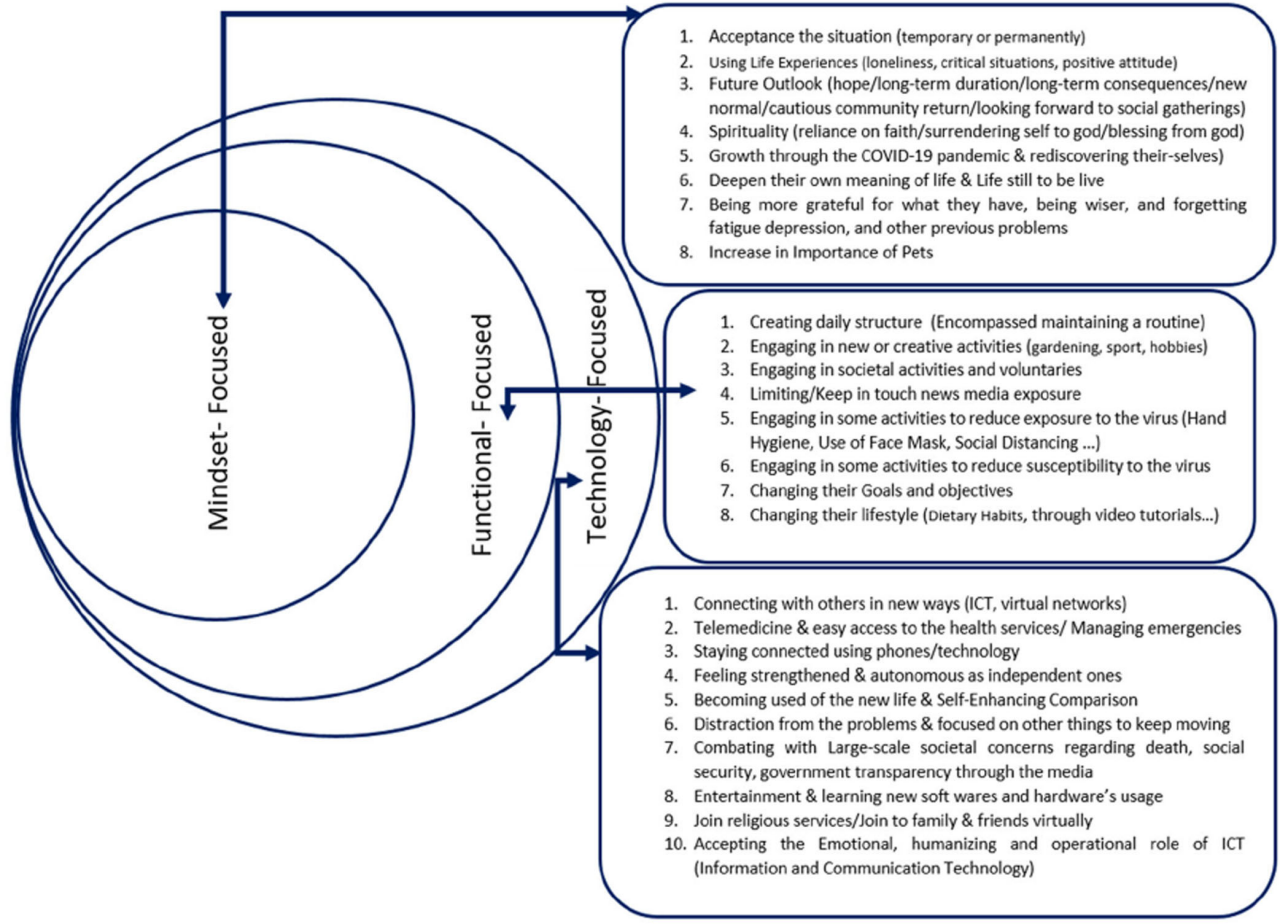
Main themes in older adults copying strategies during the COVID‐19 pandemic.

Based on the included articles, we identified two main thematic categories: (i) Retelling the expression of the emotional dimensions of the older adults during the COVID‐19 pandemic, and (ii) The ways they coped with those conditions. These categories are illustrated in Figures 2 and 3.

#### 3.3.1 Main Explored Themes of the Dimensions of the Older Adults Emotional Experiences

Being involved in the conditions of the COVID‐19 pandemic and experiencing compulsory isolation has caused many positive and negative emotional, physical, and societal conflicts among older adults individuals worldwide. Although some external factors were likely related to the level of government focus on aging, considered part of governance functions and subfunctions aiming to achieve intermediate objectives and final goals of countries, these factors include:

a. Perceptions of Government Policies and Decisions
b. Receiving Social Welfare from the Government

We have ultimately grouped these challenges into four main themes, each with its own subthemes. The first main theme was Mindset‐Challenges Engagement (psychological) with 21 subthemes; the second, Physical‐Challenges Engagement, with 7 subthemes; the third, Technological‐Challenges Engagement, with 6 subthemes; and the last, Social‐Contribution Challenges, with 16 subthemes.

##### 3.3.1.1 Mindset‐Challenges Engagement

The first common theme, observed in almost all included studies, concerned both positive and negative aspects of emotions experienced mentally and expressed by older adults participants. Almost all studies reported negative emotional effects of compulsory home isolation, including psychological feelings such as feeling like a loser, regret, loneliness, depression, disappointment, aggression, vulnerability to disease, anger, anxiety, stress, fear, reflections on friends' death due to COVID‐19, individual health conditions, and even thoughts of self‐harm and suicide in one study [44]. Other negative emotions included loss of autonomy, sadness, hopelessness, powerlessness, irritability, boredom, tiredness, and sleep disorders such as nightmares, insomnia, changes in waking hours, or staying in bed longer [27, 29, 30, 32, 33, 35, 40, 41, 43, 47, 48, 49, 50, 51, 52, 56, 57, 58, 59, 63, 65, 67].

Some studies also reported positive emotional aspects of isolation, including improved mental health, better sleep quality, viewing isolation as a responsibility, engaging in housekeeping, opportunities for reading, knitting, and doing crosswords, lack of fear of death, peacefulness, and maintaining independence [30, 31, 46, 48, 52, 55, 64].

##### 3.3.1.2 Physical‐Challenges Engagement

The second main theme concerned physical challenges faced by older adults individuals, including reduced physical activities, lifestyle changes, and worsening chronic diseases. Negative effects included limited outdoor activities, muscle weakness, balance loss, worsening symptoms of arthritis and other chronic diseases, travel restrictions, closure of older adults centers, churches, or sports facilities, weight gain, and loss of spiritual activities, along with lack of assistance in daily needs [29, 30, 35, 50, 52, 55, 56, 57, 58, 59, 60, 61, 62, 67].

However, some positive impacts were noted. In Sweden and Finland, older adults people appreciated pets for helping maintain daily routines and encouraged walking while following protective measures [46, 64]. In Wuhan, protective measures were used to reduce the burden on younger family members during limited outdoor time [58]. In the United Kingdom, Turkey, and Seattle (USA), older adults people saw isolation as an opportunity to engage more in volunteer activities, home tasks, and online exercise classes with more free time and calmness [30, 40, 65].

##### 3.3.1.3 Technological‐Challenges Engagement

Technology was considered a positive, effective, and low‐cost factor. Communication technologies introduced new living arrangement challenges and opportunities for the older adults. While some were concerned about learning new devices and uncertain futures—described as a “more robotic future”—others benefited from direct communication with loved ones via the internet, expanding social networks.

Technology enabled remote medical consultations, prescription deliveries, and frequent contact with family via WhatsApp and video calls, fostering a sense of independence [32, 35, 43, 44, 46, 55, 64]. Older adults participants increasingly used news networks to stay updated on the pandemic and became aware of neighbors' needs, restoring protective roles often lost after children leave home [32, 35, 43, 44, 46, 55, 64].

##### 3.3.1.4 Social‐Contribution Challenges

Social relations involve integration, personal identity, affect regulation, and coping through mutual assistance. Social inclusion depends on qualitative relations and shared social identity [69]. From a functionalist view, social isolation constitutes social exclusion, harming individual well‐being and social cohesion, especially among vulnerable groups like the older adults [70].

Although COVID‐19 social isolation did not worsen loneliness among previously isolated participants [31], most studies showed deprivation of social identity recognition. Reduced personal networks increased loneliness and negative psychological emotions [32, 45]. Telecommunication largely compensated for diminished social contact during isolation [31, 60], a change that likely would have taken much longer without the pandemic [48].

#### 3.3.2 Main Themes in Older Adults Coping Strategies During the COVID‐19 Pandemic

Two types of coping have been identified in the literature [71]:

1. Problem‐Focused Strategies: Active efforts to improve stressful situations.
2. Emotion‐Focused Strategies: Attempts to regulate emotional responses.

To provide a more comprehensive explanation, we expanded these into three coping types to better analyze older adults' cognitive appraisal and coping during COVID‐19:

1. Mindset‐Focused Strategy: Adjusting mindset to enhance adaptability in emotional responses.
2. Functional‐Focused Strategy: Balancing emotional responses with real‐life demands.
3. Technology‐Focused Strategy: Learning and using technology to overcome problems.

##### 3.3.2.1 Mindset‐Focused Strategies

This theme includes eight subthemes, illustrating how mindset affects older adults reactions to stress. Acceptance of the situation was identified as a crucial component for rational coping [28, 38, 53, 62]. Using accumulated life experience, positive attitudes, hope, and not feeling alone despite forced isolation were emphasized [11, 28, 37, 41, 58, 59, 64]. Future outlook, including hope and lifestyle modification, was addressed [28, 38, 68]. Spiritual reliance and faith were effective coping mechanisms [28, 30, 57, 62, 67].

In rural Canada, participants reported personal growth and rediscovery due to the pandemic [37]. Others reflected on life's meaning, overcoming fatigue and depression [35, 37, 51, 53, 68]. Scandinavians especially valued pets for providing life meaning and companionship [45, 46, 64].

##### 3.3.2.2 Functional‐Focused Strategies

This theme includes eight subthemes highlighting how balancing emotions and daily tasks aids adaptation. Studies from the Philippines and a Ph.D. thesis by Arlinde Johanna Dul identified maintaining routines as “creating a new structure” during isolation [62, 66]. Participants set goals, planned, stayed consistent, and rewarded themselves to navigate the pandemic.

Engagement in gardening, sports, hobbies, social and volunteer activities alleviated boredom and loneliness [11, 27, 28, 30, 35, 37, 39, 46, 57, 59, 62, 65]. Hygiene practices and social distancing helped reduce virus exposure [11, 27, 38, 41, 45, 57, 58, 66].

Media was a safe information source, though some limited exposure due to fake news and stress control [27, 34, 38, 43, 47, 50, 52, 58, 59, 63, 65, 67]. Participants adapted lifestyles, such as modifying diets and learning via video tutorials [30, 41, 43, 49, 52, 55, 60, 61, 63, 66].

##### 3.3.2.3 Technological‐Focused Strategies

The COVID‐19 pandemic increased the prominence of Information and Communication Technologies (ICT). Older adults participants used ICT to connect virtually with family, join religious services, access entertainment, and distract from problems [27, 29, 30, 32, 35, 37, 38, 39, 40, 41, 42, 48, 53, 56, 57, 58, 59, 62, 64, 65, 68].

Telemedicine provided comfort to lonely older adults with chronic diseases requiring constant care [33, 41, 42, 43, 44, 46, 58, 59, 65]. These technologies empowered the older adults, fostering autonomy and enabling self‐enhancement, reducing concerns about death and social security, and enhancing acceptance of ICT's emotional and operational roles [28, 35, 38, 43, 53, 56, 62, 66, 67].

## 4 Discussion

Considering the number of confirmed COVID‐19 cases and the high mortality rates reported worldwide, it is undeniable that normal life has been significantly altered by the pandemic and its consequences. The older adults, as a vulnerable group, have been particularly affected by these unexpected transformations and challenges. This systematic review aimed to explore the emotional experiences of the older adults and their coping strategies during the pandemic, based on their own words in interviews from selected studies, to provide evidence‐based insights for health policymakers facing similar future pandemics. It is important to note that throughout this review, “quarantine” refers specifically to government‐imposed stay‐at‐home measures that applied broadly, not to medical isolation of infected individuals or to general recommendations for physical distancing. This distinction helps clarify the scope of the experiences analyzed across various countries with different pandemic responses.

### 4.1 Emotional Impact

The main emotional themes extracted from the included studies reveal that although both positive and negative emotions were experienced by older adults, negative feelings predominated. These included isolation‐related emotions such as regret, loneliness, depression, hopelessness, anxiety, fear of death, irritability, boredom, fatigue, and sleep disturbances (e.g., nightmares) [27, 29, 30, 32, 33, 35, 40, 41, 43, 47, 48, 49, 50, 51, 52, 56, 57, 58, 59, 63, 65, 67]. Conversely, some participants reported adaptive positive emotions, such as improved sleep quality, viewing isolation as a responsibility, engaging in hobbies like knitting and reading, peace, and maintaining independence [30, 31, 46, 48, 52, 55, 64].

Early in the pandemic, it was expected that COVID‐19 would cause severe psychological distress among the older adults; however, emerging evidence suggests a more nuanced reality [48]. Our qualitative findings complement these results, indicating that older adults' resilience may be enhanced by factors such as digital literacy, life experience, economic security, community support, and effective coping strategies. Thus, despite initial concerns, many older adults individuals managed to adapt well to isolation and social disconnection.

### 4.2 Coping Strategies

Across the studies, older adults participants demonstrated vigilance in monitoring their health and adhered strictly to COVID‐19 preventive measures. Common coping strategies included physical activities (e.g., indoor exercise, yoga, tai chi), engagement in hobbies (knitting, cooking, gardening), maintaining a healthy diet, and limited social contacts mostly for essential needs [11, 27, 28, 30, 35, 37, 38, 39, 43, 46, 57, 59, 62, 65, 66].

The ecological, social, and cultural environment was highlighted as a critical foundation for resilience [72]. Figure 3 summarizes synergistic coping factors consistent with recent studies, even in low‐income settings such as Ghana [11, 27, 29, 31, 32, 33, 34, 35, 36, 37, 38, 42, 43, 47, 48, 53, 55, 56, 57, 58, 59, 61, 63, 64, 65, 67, 68].

Government and healthcare interventions played an important role in strengthening older adults resilience during quarantine. For example, in developed countries, home delivery of medications and telehealth services helped reduce exposure risk and provided reassurance, contributing to a sense of inclusion and the perception that the older adults have not been forgotten [29, 33, 43, 44, 48, 60, 64]. Conversely, in low‐income countries, lack of such services resulted in increased isolation and reliance on family or friends [58, 61, 62, 67]. This disparity highlights a potential digital and resource access bias in the studies reviewed.

### 4.3 Role of Technology and Social Networks

The social network of the older adults is vital for psychological well‐being, with isolation potentially leading to serious consequences, such as the case reported in Buenos Aires where loss of social bonds led to suicidal ideation [43]. ICT and artificial intelligence have played an important role in mitigating social isolation, allowing older adults to maintain contact with family and communities through platforms like Facebook, WhatsApp, Google Meet, and Skype [56, 73].

However, digital inequalities and limited digital literacy among older adults reduce the effectiveness of technology‐based interventions [74, 75]. Our findings indicate that learning and engaging with ICT can boost resilience, but not all older adults individuals have equal access or skills. Factors such as education level influence digital adoption [76, 77].

### 4.4 Vulnerable Subgroups

Certain subpopulations, such as older adults individuals living alone, minority ethnic groups, and sexual minorities (LGBT+), experienced heightened emotional vulnerability and discrimination during the pandemic [11, 33, 36, 40, 50, 51, 52, 56, 59, 64, 66]. While some studies reported that living alone may worsen emotional health, others suggested rural older adults accustomed to solitude may experience better well‐being [28, 29, 35]. Virtual communication platforms provided crucial support for marginalized groups, enabling safe and inclusive social interactions [40].

Older adults immigrants, particularly from China, faced racism and stigmatization exacerbated by the pandemic [36]. This underscores the intersectionality of emotional experiences influenced by sociodemographic factors.

Although none of the selected studies specifically addressed elder abuse, other research documents increased physical and financial abuse during the pandemic [78, 79]. Therefore, monitoring and addressing elder abuse should be prioritized by policymakers.

### 4.5 Gender Considerations

Gender did not emerge as a significant factor in emotional experiences in this review; however, the disproportionate impact of the pandemic on women, including older adults women and sexual minorities, cannot be overlooked [80, 81]. Women have faced greater burdens such as increased domestic responsibilities, exposure to violence, and job losses, all of which may influence emotional health and resilience.

### 4.6 Limitations

This study has several limitations that warrant discussion. First, as with all qualitative research, the findings are inherently context‐bound and not generalizable to broader populations. Second, the included studies primarily collected data during the first and second waves of the COVID‐19 pandemic—a period marked by widespread uncertainty and a lack of control over health‐related circumstances. This temporal focus may have biased participants' emotional responses and coping strategies, potentially underrepresenting adaptive mechanisms that could have emerged later in more stable conditions, such as after the introduction of vaccines and the expansion of hospital capacities.

Third, only a limited number of the selected studies conducted follow‐up (second‐round) interviews, which restricted the opportunity to develop a deeper rapport with participants and to capture changes in emotional experiences and coping strategies over time. Fourth, although participants came from racially diverse backgrounds, the majority were white. This demographic imbalance limited the potential to assess racial and ethnic variations in emotional responses and resilience.

Fifth, the review focused exclusively on older adults living at home or within community settings. As a result, the experiences of institutionalized older adults individuals—who may face more severe emotional and environmental adversities—were not represented, pointing to the need for further targeted research on this population. Finally, the inclusion and exclusion criteria applied in selecting the reviewed studies may have introduced selection bias, which could affect the representativeness and diversity of the synthesized findings.

## 5 Conclusion

This study provides an in‐depth comprehensive understanding of the vicissitudes in the lives of home‐dwelling older adults during unexpected pandemics like COVID‐19. According to the findings and the main themes extracted from the selected studies in this review, older adults faced psychological, physical, social, and technological challenges. Although our expectations predicted a negative emotional burden of the pandemic on this vulnerable group, our study concluded some different outcomes; surprisingly, most older adults faced the pandemic situation with a more rational and coherent understanding of their circumstances. By emphasizing their previous life experiences, they creatively organized their essential ability to adapt to the conditions; they coped intricately with these challenges by focusing on modus vivendi through mindset‐, functional‐, and technology‐focused strategies. Their unexpected approach could be considered a significant consideration for health policymakers who, considering the aging of the world's population, are looking for mechanisms to empower and enhance the resilience of older adults in future critical situations. These findings enable them to enhance the performance of their health systems by centering planning on empowering the resilience of older adults through the provision of services based on their powerful capabilities. Furthermore, as the study's results showed, underestimating their strengths, power, and abilities not only diverts the focus of health system resources from hygiene, prevention, and education toward long‐term medical care and wastes resources, but also has a destructive effect on their emotional well‐being as effective participants in their communities.

We hope that policymakers will allocate resources to more centralized resilience strategies for home/community‐dwellers who have no acute health problems and are free of cognitive and memory deficits, such as educating older adults about new technologies and new conditions and terminologies of the virtual world, and empowering their physical and emotional strength through community‐oriented recreational activities.

## Author Contributions

**Zeinab Dolatshahi:** conceptualization, investigation, funding acquisition, writing – original draft, methodology, validation, visualization, resources, formal analysis, supervision, data curation. **Pouran Raeissi:** data curation, validation. **Shahin Nargesi:** conceptualization, investigation, writing – original draft, methodology, validation, visualization, writing – review and editing, formal analysis, project administration. **Nadia Saniee:** data curation, resources. All authors read and approved the final version of the manuscript.

## Disclosure

The corresponding author Shahin Nargesi affirms that this manuscript is an honest, accurate, and transparent account of the study being reported; that no important aspects of the study have been omitted; and that any discrepancies from the study as planned (and, if relevant, registered) have been explained.

## Ethics Statement

All data and analyses in this study were based on previously published studies, thus ethical approval is not applicable for it.

## Consent

Patient consent and consent for publication are not applicable.

## Conflicts of Interest

The authors declare no conflicts of interest.

## Supporting information


**Table 2:** Characteristics of the Selected Studies.

**Table 3:** Outcomes (Themes & Sub‐Themes) of the Selected Studies.

## Data Availability

No data sets were generated or analyzed during the current study.
