# Supplementary material for: Emotional Experiences of the Home‐Dwelling Older Adults During the Isolation of the Coronavirus Disease 2019 Pandemic: A Qualitative Systematic Review
Source: Health Sci Rep. 2025 Dec 3;8(12):e71614. doi: 10.1002/hsr2.71614 (PMC12675137; doi:10.1002/hsr2.71614)
Supplement: Supplementary file 1 — Table 2: Characteristics of the Selected Studies. [file HSR2-8-e71614-s001.docx]

Table 2: Characteristics of the Selected Studies

| **First**  **Author** | **Country**  **/Publish Year** | **Study**  **Design** | **Method of Sampling** | **Mean Age** | **Gender/ (%)** | **Sample Size** | **Education** | **Time of data collection** | **Data Collection Method** | **Study Aim** | **Analyzing Method** | **Major**  **Findings** |
| --- | --- | --- | --- | --- | --- | --- | --- | --- | --- | --- | --- | --- |
| **R. Turner Goins**  **(33)** | **USA**  **2021**  **Carolina** | **qualitative descriptive**  **Study** | **convenience sampling approach** | **72.4±6.7** | **Female 55%**  **Male**  **45%** | **43** | **No Mentioned** | **25 April**  **07 May**  **2020** | **Semi structured in-depth interviews**  **by telephone** | **To understand COVID-related perceptions and behaviors of older adults residing in the United States.** | **A well-established mixed inductive, deductive, and reflexive analysis**  **analytic framework** | **Their findings underscore the importance of the preservation of mental health during extended periods of isolation by taking advantage of low-to-no-cost existing resources.** |
| **Heather R. Fuller**  **(34)** | **USA**  **2021**  **North Dakota**  **&**  **Minnesota** | **qualitative mixed methods design** | **Community-based sample** | **70-79**  **(81.6)** | **Female (72%)**  **Male**  **(28%)** | **76** | **1-30% graduate degree**  **2-30% college associate technical**  **3-17% bachelor** | **28 April**  **20 March**  **2020**  **(during early weeks of regional social distancing)** | **Semi-structured interview**  **by telephone** | **To examine levels of self-rated perceived coping among older adults, as well as explore ways older adults are coping with the sudden need to socially isolate.** | **A systematic, thematic content analysis following a framework methodology** | **Reliance on faith for coping was an important theme throughout these interviews, whether faith related activities, receiving social support from a faith-community, or practicing their faith as part of their positive mindset.** |
| **Kathy Lee**  **(35)** | **USA**  **2021**  **from urban and suburban**  **communities**  **in North Texas.** | **qualitative method** | **convenient sampling method** | **73.5±6.8** | **Female (94.4%)**    **Male (5.6%)** | **18** | **Post-high school:33.3%**  **High school:**  **22.2%**  **College degree: 17.6%**  **Graduate degree: 5.6%** | **July through**  **August 2020** | **in-depth interviews** | **To understand how COVID-19 affected the marginalized older adults mobility and daily lives.** | **A traditional**  **qualitative analytical methodology** | **Contrary to expectations**  **that they would demonstrate negative psychosocial**  **Consequences from physical distancing, most were coping well.**  **And they found no evidence of increased clinical depression, anxiety, or suicidal thoughts compared to symptoms assessed before the onset of distancing.** |
| **Mikael Anne Greenwood-Hickman**  **(36)** | **USA**  **2021**  **Seattle** | **qualitative method** | **Interview participants were invited from the Healthy Aging Resources to Thrive (HART) trial** | **60-80** | **Female (64%)**  **Male (36%)** | **25** | **67% college degree**  **29% Some college (no-degree)** | **between June and August of 2020** | **semi-structured interviews** | **To explore the physical, mental, and social health impacts of the pandemic on older adults and their coping techniques.** | **Traditional as well as innovative methods,**  **By Phone**  **an inductive approach** | **Their findings suggest a holistic approach to enhance the resilience of older adults during an unprecedented event.** |
| **Alexandra J. Fiocco**  **(40)** | **Canada**  **2021**  **Ontario** | **Descriptive**  **Qualitative Analyses** | **Snowball sampling** | **72.23±4** | **Female (59%)**  **Male (41%)** | **22** | **Not Mentioned** | **between 18 May and 01 October of 2020** | **Semi-structured one-on-one**  **open-ended interviews** | **to better understand the lived experience of community dwelling older adults during the first six months of the pandemic in Ontario, Canada** | **inductive thematic approach**  **By Phone** | **Participants reported high levels of stress and a large decrease in in-person social connection. Maintenance of a positive attitude and perspective gained from past hardships was also an important coping strategy for many participants.** |
| **Rachel V. Herron**  **(41)** | **Canada**  **2021**  **Manitoba** | **Descriptive**  **Qualitative Analyses** | **Community-based sample** | **65-89** | **Female (76.9%),**  **Male (23.1%)** | **26 community-dwelling older adults (65+) living in rural Manitoba** | **53.8% college degree**  **26.5% high school**  **11.5% under high school** | **1^st^ Interview:**  **May-June of 2020 (26)**  **2^nd^ Interview:**  **July-August**  **2020 (25)** | **semi-structured telephone**  **interviews** | **To explore older adults’ experiences of isolation and loneliness in the initial stages of the pandemic** | **inductive thematic analysis** | **Participants shared their coping strategies to maintain health and wellbeing, including behavioral strategies, emotion-focused strategies, and social support. Overall, this research highlights resilience among older adults during the first six months of the pandemic.** |
| **Juah Kim**  **(70)** | **South**  **Korea**  **2021**  **Seoul, Daejeon, Chungcheong Province** | **Descriptive qualitative study** | **snowball sampling method** | **73.69** | **Female (76.9%)**  **Male**  **(23.1%)** | **13**  **community-dwelling older adults** | **Participants were most often high-school graduates** | **between November 2020 and February 2021** | **In-depth interviews** | **To examined the effects of strong quarantine measures and social distancing on older adults’ lifestyles.** | **qualitative thematic analysis** | **Our findings call attention to the role of different environments and resources in supporting older adult’s social and emotional wellbeing, particularly as they adapt to changes in social contact over time.** |
| **Avinash Chakrawarty**  **(47)** | **India**  **2021** | **Mixed Qualitative Study** | **snowball sampling technique** | **62.2** | **Female**  **(45.4%)**  **Male**  **(54.6%)** | **13** | **Not Mentioned** | **Sep 2020** | **7 In-depth Interview&**  **3 Focus Group(FGDs)**  **(5 ones/per group)** | **To explore psychosocial and behavioral impact of COVID‑19 on the lives of the older adults.** | **conventional content analysis method** | **They expressed fear about COVID-19 infection and anxiety about COVID-19-related news, and they often felt bored and depressed. Non-face-to-face community support is urgently needed for older adults facing reduced levels of physical activity and psychological hardships due to the covid-19 pandemic.** |
| **Regina W.-S. Sit**  **(54)** | **Hong Kong**  **2021** | **qualitative study** | **Theoretical purposive sampling technique** | **72.6** | **Female**  **(95.7%)**  **Male**  **(4.3%)** | **23** | **Not Mentioned** | **April 2020** | **Semi-structured in-depth telephone interviews** | **To explain the psychosocial effects of COVID-19 on Hong Kong Chinese older people.** | **Inductive Thematic analysis method** | **Elderly people are affected in many ways due to COVID‑19. Addressing the psycho‑social and behavioral problems can help in the better adjustment to tide over the pandemic** |
| **Qing Yang**  **(15)** | **China**  **2021**  **Wuhan** | **An empirical phenomenological approach**  **Of qualitative study** | **purposive sampling** | **72±5.5** | **Female**  **(55.6%)**  **Male**  **(44.4%)** | **18** | **5.6% college degree**  **38.9% high school**  **27.8% under high school** | **July 24 and August 2, 2020** | **semi-structured, in-depth telephone interviews** | **To explore the experiences of community-dwelling older adults in Wuhan during the coronavirus disease 2019 lockdown.** | **Grounded**  **theory** | **Self-isolation has disproportionately affected older individuals whose only social contact is out of the home. Online technologies can be harnessed to provide social support networks and a sense of belonging, but its adaptive and positive uses should be encouraged.** |
| **Miriam**  **Verhage**  **(60)** | **Netherlands**  **2020**  **Leiden** | **qualitative study** | **snowball sampling** | **75.5** | **Female**  **(57.6%)**  **Male**  **(42.4%)** | **59** | **Not Mentioned** | **27 March and 20 April 2020** | **Semi**  **structured telephone interviews** | **To explore how Dutch older adults view this crisis and cope with measures to contribute to our understanding of coping of older adults in general and during disaster situations more specifically.** | **a computer-assisted qualitative data software MAXQDA** | **Our findings stress the importance of acknowledging heterogeneity among older adults and adjusting communication about mitigation measures to decrease insecurity and increase resonance. This may make COVID-19 mitigation measures more manageable and age responsible and allow older adults to start living again** |
| **Eneritz Jiménez-Etxebarria**  **(62)** | **Northern Spain**  **2021** | **qualitative study** | **Convenience sampling** | **68-81** | **Female**  **(76.9%)**  **Male**  **(23.1%)** | **26** | **42.3% higher**  **Degree**  **15.4% high school**  **42.3% under high school** | **March 2020** | **Semi**  **structured telephone interviews** | **To concern with exploring the perspective of people over 67 years of age in relation to the changes experienced in their lives, the perception of the treatment of older adults during the pandemic, their attitude towards tackling the situation resulting from the pandemic, and their expectations for the future.** | **An inductive grounded theory**  **the NVivo software tool** | **It is concluded that the perception of vulnerability that we have towards older people is erroneous, since they have been shown to have the capacity to display resilience and strength in the face of adversity.** |
| **Joanne Brooke**  **(44)** | **UK & the Republic of Ireland**  **2020**  **Birmingham** | **An inductive phenomenological study** | **Convenience sampling** | **77±5.77** | **Female**  **(68.4%)**  **Male**  **(31.6%)** | **19** | **Not Mentioned** | **during the first two weeks of household isolation** | **Qualitative semi-structured interviews** | **To explore older people's initial experience of household isolation, social distancing and shielding, and the plans they constructed to support them through the COVID-19 pandemic.** | **Theoretical framework**  **content analysis** | **Understanding the holistic life view of older people, including death anxiety, is an important element of care planning; to help older people access the protective resources, they need to reduce the serious risks associate.** |
| **A. R. McKinlay**  **(45)** | **UK**  **2021** | **qualitative Study** | **Purposive sampling methods** | **70-90** | **-** | **20** | **Not Mentioned** | **March 2020** | **Semi-structured telephone or video interviews** | **To examine factors that threatened and protected the wellbeing of older adults living in the UK during social distancing restrictions due to the COVID-19 pandemic.** | **reflexive thematic analysis** | **While some older adults experienced challenges during the first wave of COVID-19, many were resilient throughout social distancing restrictions despite early reported concerns of mental health consequences among the older adult population. The findings highlight the importance of maintaining access to essentials to promote feelings of normality and use of social support to help reduce uncertainty in times of pandemics.** |
| **Henry**  **Bundy**  **(37)** | **North Carolina, USA**  **2021** | **qualitative study** | **convenience sampling, plus a non-probability sampling strategy** | **65-92**  **(75)** | **Female**  **(58.3%)**  **Male**  **(41.6%)** | **12** | **No Mentioned** | **2020** | **semi -structured interviews** | **To understand how already-lonely older individuals navigated and endured the social isolation of the pandemic.** | **inductive—building**  **theory** | **Found that for some already-lonely older adults, the pandemic had reconstituted their conception of loneliness and that the loneliness experienced by our interviewees was not additive; that is, the distress of being alone was not exacerbated by social distancing and stay-at-home orders.** |
| **Akira Teramura**  **(67)** | **Japan**  **2021**  **Kyoto City** | **qualitative inductive research method** | **snowball sampling** | **75-85** | **Female**  **(77%)**  **Male**  **(23%)** | **13** | **15.3% Postgraduate**  **23% undergraduate**  **23% High School**  **7.6% College degree** | **from 20 March to 29 April 2021** | **semi -structured interviews** | **To understand COVID-19-related lifestyle changes experienced by older adults who lived in communities and used day-care services.** | **qualitative descriptive analysis**  **and a computer-assisted qualitative data software MAXQDA** | **Indicate that the closure of public places and the fear of infection during the COVID-19 pandemic caused not only a decrease in human contact, but also a decrease in activities and a disruption of daily life rhythms among older adults, along with a negative impact on their physical and mental functioning.** |
| **Pedro**  **Pisula**  **(50)** | **Argentina**  **2021**  **Buenos Aires** | **exploratory qualitative study** | **Snowball sampling** | **67-76**  **73** | **Female**  **(82%)**  **Male**  **(18%)** | **39** | **41% Secondary**  **26% high school**  **18% Primary School**  **15% No data** | **April and July 2020** | **Semi-structured telephone or video interviews** | **To explore the emerging needs related to the mental health of isolated older adults in this period and to identify their main support networks they have and the emerging coping strategies in the face of the situation.** | **Inductive conceptual frameworks**  **Atlas.ti 8 software was used for coding.** | **Identified greater vulnerability in people living alone, in small and closed environments, with weak linkages and networks, or limited access to technologies. And also found various coping strategies and technology was a fundamental factor in maintaining the bonds.** |
| **Candela Agustina Loza**  **(51)** | **Argentina**  **2021**  **Buenos Aires** | **exploratory qualitative study** | **Snowball sampling** | **67-76**  **73** | **Female**  **(82%)**  **Male**  **(18%)** | **39** | **41% Secondary**  **26% high school**  **18% Primary School**  **15% No data** | **Not Mentioned** | **Semi-structured telephone interviews** | **To explore the elderly’s healthcare experiences during the lockdown and the problems that may have arisen regarding accessibility to the healthcare system and emerging adaptations to medical care.** | **Inductive conceptual frameworks**  **Atlas.ti 8 software was used for coding.** | **Detected poor quality information associated with fear and anxiety and led to avoidance behaviors when consulting the health system.** |
| **Heather R. Fuller &**  **Andrea**  **Huseth-Zosel**  **(38)** | **USA**  **2021**  **North**  **Dakota & Minnesota** | **mixed-method** | **convenience**  **sampling** | **70–97**  **82** | **Female**  **(74%)**  **Male**  **(26%)** | **76** | **In Rural Area: 13.8 years on education**  **In Non-Rural Area: 15.5 years on education** | **March 28 and April 20, 2020** | **Semi-structured telephone interviews** | **To examine changes in older adults’ loneliness due to social distancing, explore variability in perceptions, and identify whether such changes differ by rurality** | **quantitative analyses: SPSS Version 26.0**  **Qualitative analysis: preliminary analysis.** | **Findings indicate increases in older adults’ loneliness during early weeks of the pandemic. While these findings suggest nuanced experiences among older adults, it is imperative to track the implications of isolation and loneliness due to social distancing over time among diverse samples of older adults.** |
| **Kulmala J**  **(52)** | **Finland**  **2020** | **qualitative study** | **Purposive sampling methods** | **84.8** | **Female**  **(66.7%)**  **Male**  **(33.3%)** | **15** | **Not Mentioned** | **August 20, and December 1-2020** | **Semi-structured interviews** | **To gain a more in-depth understanding of how life in the shadow of a global pandemic affected personal social networks among the oldest old communitydwelling people.** | **directed**  **content**  **analysis** | **Social activity and personal networks play an important role in the well-being of the oldest old, but individual situations, needs, and preferences toward personal social networks should be taken into account when planning social activities, policies, and interventions.** |
| **Tiilikainen E**  **(53)** | **Finland**  **2021** | **mixed-**  **methods**  **approach** | **Purposive sampling methods** | **84.8** | **Female**  **(66.7%)**  **Male**  **(33.3%)** | **15** | **Not Mentioned** | **between**  **August and December**  **2021** | **telephone interviews** | **To support older people’s meaningfulness in challenging times.** | **thematic**  **analysis** | **Findings show that despite protective measures, it is important to ensure that older people have the possibility to maintain self-determination and make decisions regarding one’s everyday life. Moreover, the findings highlight supportive factors in the oldest old’s daily life, as well as their own ways of pursuing meaningfulness in challenging times.** |
| **Siu‑Ming Chan**  **(55)** | **Hong Kog**  **2022** | **mixed-**  **methods**  **approach** | **Purposeful sampling** | **60-91**  **71.4** | **Female**  **(50%)**  **Male**  **(50%)** | **10** | **Upper secondary 10%**  **High School 30%**  **Junior High 10%**  **Junior School 20%**  **Primary or below 30%** | **Between**  **September 2020 and April 2021** | **in-depth**  **semi-structured qualitative**  **interviews** | **To examine the psychosocial vulnerability of older adults relative to their younger counterparts and explore how they cope with the pandemic.** | **thematic**  **analysis** | **Older adults in this study showed better psychosocial well-being than their younger counterparts under the COVID-19 pandemic, which challenged the deeply rooted societal stereotype about the vulnerability of older adults. The stronger resilience for positive coping, technological assistance, and targeted government and com‑ munity support may have protected older adults from distress during the pandemic.** |
| **Pranab Mahapatra**  **(48)** | **India**  **2021** | **A reflective narrative**  **approach** | **Purposive sampling** | **60-85**  **67** | **Female**  **(50%)**  **Male**  **(50%)** | **11**  **couples** | **Not Mentioned** | **during the last week of June to the middle of July 2020** | **Telephonic**  **in-depth**  **semi-structured interviews** | **To explore the ‘coping reflections’ of elderly couples living alone (without any other family members) during the COVID-19 pandemic in urban Odisha, India.** | **inductive**  **approach of thematic**  **approach**  **analysis**  **(MAXQDA**  **Analytics Pro 2020)** | **Findings indicate spousal support and social network with adaptive organizational change and a responsive public system to be crucial in mitigating the older adults’ challenges in a pandemic.** |
| **Rasoul Norouzi Seyed Hosseini**  **(56)** | **Iran**  **Tehran**  **2021** | **A phenomenological**  **approach** | **Purposive sampling** | **Over 60** | **Female**  **(0%)**  **Male**  **(100%)** | **16** | **Not Mentioned** | **from March 11, 2019, to May 20, 2020** | **in-depth and**  **unstructured**  **interviews** | **To understand the lived experience of athletic older adults from the Coronavirus Disease 2019 (COVID-19) pandemic.** | **The seven-step Diekelman’s (1993)**  **Approach.** | **Older athletes have attempted to adapt to the new conditions and develop creativity to have the desired physical activity. They have experienced active and creative activism in sports, resulting from the transformation of threats into opportunities and the acceptance of a lifestyle in the context of the COVID-19 pandemic.** |
| **Mohammad**  **Asgari**  **(57)** | **Iran**  **Tehran**  **2021** | **A Phenomenological Study** | **Purposive sampling method** | **66-75**  **69** | **Female**  **(47%)**  **Male**  **(53%)** | **15** | **Junior High School 7%**  **Upper High School 27%**  **Bachelor of Science 40%**  **Master of Science 26%** | **2020** | **semi-structured interviews** | **To investigate the psychological experiences of the elderly during the pandemic of COVID19.** | **content analysis method with Colaizzi**  **analysis** | **The prevalence of COVID-19 has strongly affected all aspects of mental health in the elderly and that the serious effects of this pandemic have influenced the various aspects of their individual, interpersonal and social life. So the elderly face many challenges during the outbreak of COVID-19 and need to receive appropriate psychological interventions.** |
| **Jonaid M. Sadang**  **(69)** | **Philippines**  **Marawi City**  **2021** | **A qualitative instrumental case study** | **Purposive and**  **Snowball**  **sampling** | **Over 65**  **69** | **Female**  **(80%)**  **Male**  **(20%)** | **5** | **Not Mentioned** | **between March and December 2020** | **In-depth**  **interviews** | **To validate one of the propositions of the Need-Threat Internal Resiliency Theory.** | **a phenomenological holistic description** | **Older adult participants had established internal resiliency in response to changes caused by the COVID-19 crisis, resulting in adaptation and coping with the situation, consistent with one proposition of the Need-Threat Internal Resiliency Theory.** |
| **Ilaria Falvo**  **(58)** | **Switzerland**  **in the**  **Italian-speaking region** | **An interpretive (hermeneutic) phenomenological approach** | **convenience and snowball**  **sampling** | **aged 64+**  **75** | **Female**  **(73%)**  **Male**  **(27%)** | **19** | **Primary 74%**  **Secondary 16%**  **University 5%** | **between Apr 2 and May 15, 2020** | **semi-structured phone interviews** | **To explore the lived experiences of individuals aged 64 or older during the first COVID-19 lockdown** | **The six-stage comprehensive thematic**  **analysis**  **approach (Braun and Clarke)** | **They found a high degree of ambivalence experienced by our participants at the individual, micro-, meso-, and macro-social levels, and discovered that, by recognizing all older adults as an at-risk category, the current pandemic has the potential to change the social representation of old age and reinforce ageism-based beliefs and attitudes among the population.** |
| **Sabrina Cipolletta**  **(59)** | **Italy**  **(Northern)** | **A qualitative approach** | **snowball**  **sampling** | **72-94**  **80** | **Female**  **(64%)**  **Male**  **(36%)** | **28** | **Not Mentioned** | **between the 27th of March and 15th of April 2020** | **semi-structured phone interviews** | **To understand the experiences of isolation and strategies used to cope with it among older people living at home during the first wave of the COVID-19 pandemic.** | **The thematic analysis** | **Given the impact of social isolation on older people’s well-being, it is critical to identify and strengthen personal resources and social support strategies that may help older people cope with the restrictions imposed by the COVID-19 pandemic.** |
| **Trish**  **Hafford-Letchfield**  **(46)** | **UK** | **Mix Method** | **convenience**  **sample** | **60-79**  **In**  **lesbian, gay,**  **bisexual and trans (LGBT+) older**  **people** | **Female**  **(70%)**  **Male**  **(30%)** | **17** | **Not Mentioned** | **between 1 June and 7**  **August 2020** | **Virtual semi- structured**  **Interviews**  **(telephone, or online video-conferencing)** | **To explore the strategies used to manage by lesbian, gay, bisexual and trans (LGBT+) older people’s situations, how they responded and adapted to key challenges.** | **The thematic analysis** | **The findings illuminate adaptability and many strengths in relation to affective equality and reciprocal love, care and support among LGBT+ older people. It is vital UK that the government recognizes and addresses the needs and concerns of LGBT+ older people during emergencies.** |
| **Evi M. Kremers**  **(61)** | **Netherlands**  **(Nijmegen)** | **A qualitative approach** | **Purposive sampling** | **60-79**  **72** | **Female**  **(55%)**  **Male**  **(45%)** | **20** | **University 40%**  **Secondary School 40%**  **Elementary Technical School 15%**  **Primary School 5%** | **from 29 April 2020 until 25 June 2020** | **semi-structured phone interviews** | **To explore independently living older adults’ perceptions of social and emotional well-being during the COVID-19-related self-isolation, and their motivation to expand their social network in the future.** | **Inductive conceptual frameworks**  **Atlas.ti (version 8.4.24)** | **The relatively vital community-dwelling older adults in this study were able to adapt to the government recommendations for self-isolation with limited negative impact on their socio-emotional well-being.** |
| **Carmen Llorente-Barroso**  **(63)** | **Spain**  **Madrid** | **qualitative exploration**  **Method** | **non-probabilistic convenience sample** | **60-77**  **67** | **Female**  **(66%)**  **Male**  **(34%)** | **27** | **Ph.D. degree 11%**  **Graduate 41%**  **Certified 8%**  **Secondary 22%**  **Primary 18%** | **Not Mentioned** | **Four focus groups-** **online**  **&**  **A semi-structured interview** | **To understand the impact of the use of ICT on the emotional well-being of elderly people during their confinement.** | **inductive content analysis**  **ATLAS.ti 9 software** | **ICT have become a valuable ally for elderly people aged 60 years and older to mitigate the negative effects of social isolation and loneliness imposed by the confinement.** |
| **Jessica R Daly**  **(39)** | **USA**  **Southern California** | **qualitative study** | **Participants of the longitudinal study** | **65-100**  **80** | **Female**  **(67%)**  **Male**  **(33%)** | **21** | **81% had attained an associate degree or higher** | **During May 2020** | **Four focus groups- online**  **&**  **A semi-structured interview** | **To assess the impact of the COVID-19 pandemic and the resulting “Stay-at-Home” order on the mental and physical health of older adults and to explore ways to safely increase social connectedness among them.** | **a traditional content thematic analysis process** | **Findings suggest that technology access, connectivity, and literacy are potential game-changers to supporting the mental and physical health of older adults and must be prioritized for future research.** |
| **Qianyun Wang**  **(65)** | **Canada**  **Chinese Immigrants** | **exploratory qualitative study** | **criterion sampling**  **&**  **purposive sampling** | **65 to 83**  **73**  **Lived in Calgary for at least ten years** | **Female**  **(53%)**  **Male**  **(47%)** | **15** | **Not Mentioned** | **between late April and early May in 2020** | **In-Depth Interviews** | **To understand the unique experiences of older Chinese adults in Canada in the early stages of the COVID-19 pandemic.** | **Thematic analysis technique** | **Findings reinforce the need for anti-ageism, anti-racism and strength based social work practice, research, and policies aimed at improving older immigrants’ lives during pandemics.** |
| **Arlinde Johanna Dul**  **(16)** | **the Northern Netherlands**  **the provinces of Friesland, Groningen, Drenthe, and the north of Overijssel** | **A qualitative research** | **follow-up interviews with**  **participants who already had been included in the**  **Meaningful**  **Mobility**  **project** | **60-75** | **Female**  **(41%)**  **Male**  **(59%)** | **17** | **Not Mentioned** | **In April and May 2020** | **semi-structured phone & live**  **interviews** | **To explore the process of change and adaptation in mobility patterns and experiences during the COVID-19 outbreak and ILD measures in the Netherlands.** | **An informed grounded theory approach**  **The software program ATLAS.ti.** | **Findings show the elderly demonstrated flexibility, creativity, and the ability to adapt in the COVID-19 situation. And to combat these challenges, older adults used the adaptation strategies of selection, optimization, and compensation to maintain quality of life.** |
| **Maria**  **Alice**  **Cavacante Gomes**  **(64)** | **Brazil** | **A qualitative research** | **convenience**  **sample** | **60-79** | **Not**  **Mentioned** | **14** | **Illiterate 7%**  **Secondary 14%**  **Incomplete**  **Elementary 50%**  **Complete**  **Elementary 29%** | **in July 2020** | **semi-structured interviews** | **To unveil the experience of the elderly with social isolation in the pandemic of COVID-19.** | **The software Interface de R pour les Analyses Multi**  **Dimensionless de Texts et de Questionnaires (IRAMUTEQ) 0.7 Alpha 2.3.3.1. Multivariate analysis** | **The experience of the elderly was permeated by the adaptation of routine, adoption of preventive measures and feelings of anguish in the face of uncertainties.** |
| **Ahmet Kosar**  **(72)** | **Turkey** | **A qualitative research** | **convenience**  **sample** | **65-74**  **64** | **Female**  **(58%)**  **Male**  **(42%)** | **12** | **University degree 50%**  **Secondary 25%**  **Primary 25%** | **twice between May and June 2020** | **semi-structured interviews** | **To reveal the vital difficulties experienced by those in the 65-75 age group, who were quarantined due to the Covid-19 global pandemic and were deprived of their old normal habits.** | **the Grounded Theory methodology** | **Three types of uncertainty, namely "ambage", "ambiguity" and "contingency", were observed concretely during the whole study.** |
| **Sofi Fristedt**  **(71)** | **Sweden** | **A qualitative research** | **From a qualitative longitudinal project.** | **Over 70**  **76** | **Female**  **(65%)**  **Male**  **(35%)** | **17** | **University**  **Education 53%**  **Post-secondary education 12%**  **Upper secondary education 24%**  **Lower secondary education 11%** | **in April 2020** | **remote semi-structured interviews** | **To explore how adults 70+ experienced and managed changes in everyday life due to the COVID-19 pandemic and how those changes affected wellbeing at the beginning of the virus outbreak.** | **qualitative content analysis**  **&**  **The NVivo 12 software** | **The participants questioned previous conceptions of meaning in relation to habitual activities, likely leading to consistent occupational changes. However, these long-term effects remain to be explored, and considered to enable older adult’s health during the pandemic and beyond.** |
| **Paxton Bruce**  **(43)** | **Canada** | **qualitative descriptive approach** | **Participants from the last study** | **64-72**  **68.5** | **Female**  **(80%)**  **Male**  **(20%)** | **55** | **Not Mentioned** | **from April of 2020 to September of 2020** | **semi-structured interview** | **To investigate the elderly’s experiences during the COVID-19 pandemic and the effects on the volunteer–client relationship.** | **inductive thematic coding** | **Findings provide important insights into the experiences of the elderly volunteers and clients during the COVID 19 pandemic and the importance of acknowledging both older persons’ vulnerability and their resilience, finding innovative ways to foster volunteer–client relationships during times when physical visiting is not possible.** |
| **Moumita Das**  **(49)** | **India**  **in West Bengal** | **a qualitative study** | **Purposive**  **sampling** | **>60** | **Female**  **(60%)**  **Male**  **(40%)** | **15** | **University**  **Education 20%**  **Secondary 33&**  **Primary 47%** | **from April 7th, 2020 to April 14th, 2020** | **semi-structured interview** | **To identify the experiences of social distancing on their lives and their situation in an everyday living context and to develop plans and policies towards awareness, counseling, and volunteering services based on local or remote area approach to make coping strategies for them more viable to combat this unforeseen crisis.** | **qualitative content analysis** | **Social isolation and distancing is a major strategy to reduce the chance of infection in them that in turn resulted in major psychological implications such as loneliness, depression, and anxiety** |
| **Prince Chiagozie Ekoh**  **(68)** | **Nigeria**  **a rural area in Awgu**  **Local Government Area of Enugu State, southeast Nigeria** | **a qualitative study** | **Simple**  **random sampling** | **60-81** | **Female**  **(64%)**  **Male**  **(36%)** | **11** | **University**  **Education 10%**  **Secondary 18&**  **Primary 36%**  **No formal**  **Education 36%** | **Not Mentioned** | **in-depth interviews** | **To investigate the impact of the COVID-19 pandemic on rural older people in Nigeria.** | **Nvivo12 and analyzed thematically.** | **Social workers should therefore advocate the distribution of food and care supplies to rural older people to cushion the economic impact of diminishing social support, and also creatively help them maintain social connectedness.** |
| **Delali**  **Adjoa Dovie**  **(66)** | **Ghana** | **An**  **interpretive phenomenology research** | **Purposive**  **sampling** | **60-84**  **71.5** | **Female**  **(50%)**  **Male**  **(50%)** | **10** | **Doctorate degree 10%**  **Diploma 20%**  **Middle School leaver 40%**  **First degree 30%** | **between March and October, 2020** | **semi-structured interview** | **To investigate older Ghanaian adults’ lived experiences of the COVID-19 pandemic along the trajectory of social, healthcare, childcare, self-care and humanitarian dimensions and how they navigated the effects.** | **Qualitative analysis proposed by**  **Collaizzi’s phenomenological method with NVivo software version 11.** | **Loneliness and long-term social distancing physiologically have the propensity to decrease the ability of an individual to fight infections and inflammations.** |
| **Kathleen Melei**  **(73)** | **Croatia** | **a qualitative study** | **convenience**  **sampling** | **65 or older**  **73.4** | **Female**  **(80%)**  **Male**  **(20%)** | **5** | **Not Mentioned** | **between January and February 2021** | **semi-structured interview** | **To explore experiences of social isolation and loneliness during the pandemic.** | **Thematic analysis technique**  **Based on Model of Human Occupation (MOHO: Model** **with 4 main constructs that explain a person's view of self and their priorities: volition, habituation, performance capacity, and environment -)** | **Findings revealed a direct and interdependent relationship between social participation and quality of life. The pandemic also resulted in a complete loss of experiences as individuals reported several cancelled vacations, retirement plans, leisure activities, and even family rituals.** **The participants indicated feelings of uncertainty, frustration, disillusionment, and even disengagement because of online conflict, disappointment in leadership, and uncertainty regarding the rules.** |
| **Salina Jivan**  **(74)** | **USA**  **From different states** | **a qualitative study** | **convenience**  **sampling** | **65 years or older** | **Female**  **(74%)**  **Male**  **(26%)** | **42** | **Not Mentioned** | **May 2020** | **semi-structured interview** | **To understand the experiences of social distancing on older adults’ lives.** | **thematic analysis** | **The long-lasting effects of social distancing on mental, physical and social health are unknown at this time, but these senior participants suggested it would have a lasting toll on their emotional wellbeing and desire for social connection.** |
