# Supplementary material for: Emotional Experiences of the Home‐Dwelling Older Adults During the Isolation of the Coronavirus Disease 2019 Pandemic: A Qualitative Systematic Review
Source: Health Sci Rep. 2025 Dec 3;8(12):e71614. doi: 10.1002/hsr2.71614 (PMC12675137; doi:10.1002/hsr2.71614)
Supplement: Supplementary file 2 — Table 3: Outcomes (Themes & Sub‐Themes) of the Selected Studies. [file HSR2-8-e71614-s002.docx]

| 1^st^ Author/Country | Themes | | | Sub-Themes | |
| --- | --- | --- | --- | --- | --- |
|  | Type | Positive | Negative | Positive | Negative |
| R. Turner Goins  USA  (Carolina)  (33) | Emotional | Risk Perception | Risk Perception | No consider themselves in a ‘high risk category’ | Consider themselves in a ‘high risk category’ |
|  |  | Financial Impact | Financial Impact | The pandemic has not impacted their financial situation | The pandemic has impacted their financial situation |
|  |  | Emotions | - | Not Affected | Affected (Anxiety- Fear- Loneliness) |
|  |  |  |  |  | Uncertainty about the future |
|  |  |  |  |  | Disappointment |
|  |  |  |  | Positive Feelings | - |
|  | Coping | Problem-Focused | - | Engaging in some activities to reduce exposure to the virus | - |
|  |  |  |  | Engaging in some activities to reduce susceptibility to the virus (healthier eating, meditating, exercising, and taking supplements to boost their immune system) | - |
|  |  | Emotion-Focused | - | Creating daily structure | - |
|  |  |  |  | Engaging in new or creative activities | - |
|  |  |  |  | limiting news media exposure |  |
|  |  |  |  | Connecting with others in new ways | - |
| Heather R. Fuller  USA  (North Dakota & Minnesota)  (34) | Emotional | - | - | - |  |
|  | Coping | Staying Busy | - | Engaging in Activities | - |
|  |  |  |  | Engagement in Projects | - |
|  |  |  |  | Encompassed maintaining a routine | - |
|  |  | Seeking Social Support | - | Relationship with family & Friends & community | - |
|  |  |  |  | Using social Technology | - |
|  |  |  |  | Adapting to communicating at a distance | - |
|  |  | Most of Participants Having a Positive Mindset | Some of Participants Having a Negative Mindset | Acceptance the situation | - |
|  |  |  |  | Life Experiences | - |
|  |  |  |  | Future Outlook | - |
|  |  |  |  | Reliance on Faith | - |

Table 3: Outcomes (Themes & Sub-Themes) of the Selected Studies

| Kathy Lee  USA  (from urban and suburban  communities  in North Texas)  (35) | Emotional | - | Sedentary Behaviors | - | | | Limitation Going out |
| --- | --- | --- | --- | --- | --- | --- | --- |
|  |  |  |  | - | | | Loss of Muscle Strength |
|  |  |  |  | - | | | Loss of Balance |
|  |  |  |  | - | | | Insufficient Physical Activities |
|  |  |  |  | - | | | Worsen the symptoms of chronic disease like arthritis |
|  |  | - | Mental and Social Well-being | - | | | Limited Physical Contact with Others |
|  |  |  |  | - | | | Feeling Loneliness |
|  |  |  |  | - | | | Feeling Vulnerable |
|  |  |  |  | - | | | Feeling Depressed |
|  |  |  |  | - | | | No assistance for daily needs |
|  | Coping | Individual Environment | - | Many of Participants  Using Technology | | | A few of Participants  Prior Competence |
|  |  |  |  | Join Religious Services | Join to Family & Friends | Telemedicine |  |
|  |  | Social Environment | - | Formal Support (community organization) | | |  |
|  |  |  | - | Informal Support (family & Neighbors) | | | - |
|  |  | Built Environment | - | Walkable Neighborhood | | | - |

| Mikael Anne Greenwood-Hickman  USA  (Seattle)  (36) | Emotional | - | Daily Life | - | Stay at Home |
| --- | --- | --- | --- | --- | --- |
|  |  |  |  | - | Travel Limitations |
|  |  |  |  | - | Work Challenges |
|  |  |  |  | - | Finance (Loss of Income) |
|  |  |  |  | - | Policy Impact to Behavior |
|  |  | - | Health Impacts | - | Mental Health, Energy, and Stress |
|  |  |  |  | - | Nutrition |
|  |  | Physical Activities | - | More Free Time to Be Active | Closed Exercise Facilities |
|  |  | - | Sedentary Time | - | Being Stuck at Home |
|  |  | - | Sleep Quality | Improved Sleep | Difficulties with Sleeping |
|  |  | - | Sickness/Infection with Covid-19 | - | Fear of Being Sickness or Infected |
|  |  | - | Social Impacts | - | Limitation in In-Person Connection |
|  |  |  |  | - | Limitation in Family connection (Events) |
|  | Coping | Social Connections | - | Virtual Unique Opportunities to be Engaged | - |
|  |  |  | - | Reliance on Spouse/Partner in home Connection | - |
|  |  | Activities | - | Hobbies (TV, Crafts, Reading, Gardening…) | - |
|  |  |  | - | Exercise (Fitness Online Classes, and etc.) | - |
|  |  |  | - | Following Public Health Guidance | - |
|  |  |  | - | Following HART Program | - |
|  |  | Belief | - | Positive Attitude | - |
|  |  |  | - | Spirituality | - |

| Alexandra J. Fiocco  Canada  (Ontario)  (40) | Emotional | Challenges Associated with the Pandemic | Threat Associated with the Pandemic | Challenges with physical & social distancing | Challenges with physical & social distancing |
| --- | --- | --- | --- | --- | --- |
|  |  |  |  | Challenges with health management & service | Challenges with health management & service |
|  |  |  |  | Living Arrangement Challenges | Living Arrangement Challenges |
|  |  |  |  | Use of Technology | Fear messaging in the Media |
|  |  |  |  | - | Threat of contracting the Virus |
|  |  |  |  | - | Financial Threat |
|  | Coping | Coping with the Covid-19 Pandemic | - | Emotion-Focused Strategies | - |
|  |  |  | - | Social Support | - |
|  |  |  | - | Behavioral Strategies | - |
| Rachel V. Herron  Canada  (Manitoba)  (41) | Emotional | - | Loss of Autonomy | Perception of Risk | Fearing of individual health conditions |
|  |  | - | Loss of Social Activities | - | Boring and Lonely |
|  |  | - | Lack of Meaningful Connection at Home | - | Increased Mobility Limitations |
|  | Coping | Past Experiences of Isolation | - | Attributing Isolation to their Rural Context | - |
|  |  | Negotiating Physically Distanced Visits & Connecting | - | Outdoor visiting with social distancing roles  & Using Technology Opportunities | - |
|  |  | Keeping Busy | - | Doing Household Chores & Hobbies & Gardening | - |

| Juah Kim  South Korea  (Seoul & Daejeon & Chungcheong Province)  (70) | Emotional | - | Increased Cautiousness in daily life | - | Increased Cautions toward Others |
| --- | --- | --- | --- | --- | --- |
|  |  |  |  | - | Avoiding Objects Interacted with by Others |
|  |  | - | Psychological Changes | - | Boredom & Depression |
|  |  |  |  | - | Fear of Infection |
|  |  |  |  | - | Increased Anxiety (Media & Text Alerts) |
|  |  |  |  | - | Anger toward Others (do not follow quarantine rules) |
|  | Coping | Lifestyle Changes | Lifestyle Changes | Following Quarantine Roles in daily life | - |
|  |  |  |  | - | Increased Time Spent at home |
|  |  |  |  | - | Smaller Radius of daily Activity |
|  |  |  |  | - | Changes in Exercise Patterns |
|  |  |  |  | Changes in Dietary Habits | Changes in Dietary Habits |
|  |  | Adaption to the New Life | - | Enduring & Coming to Terms | - |
|  |  |  |  | Becoming Used of the new life | - |
| Avinash Chakrawarty  India  (47) | Emotional | - | Psychological Changes | - | Felling Fear (Getting Infected, Being Vulnerable, Going Out, Confusion) |
|  |  |  |  | - | Feeling Anxiety |
|  |  | - | Social Challenges | - | Household Confinement |
|  |  |  |  | - | Social Boycott |
|  |  |  |  | - | Inability to meet friends and family |
|  |  |  |  | - | Inability to attend social gathering |
|  |  |  |  | - | Inability to attend religious ceremonies |
|  |  |  |  | - | Inability to visit doctors/going hospitals |
|  | Coping | Behavioral Changes | - | Positive Lifestyle Modifications | - |
|  |  |  |  | Preventive Practices (Hand Hygiene, Use of Face Mask, Social Distancing …) | - |
|  |  |  |  | Spending Quality Time with Family | - |
|  |  |  |  | Usage of Social Time | - |
|  |  |  |  | Indulgence in Hobbies | - |
| Regina W.-S. Sit  Hong Kong  (54) | Emotional | - | Psychologically Unbridled Uncertainty | - | Feeling of Fear, Hopelessness, and Powerless |
|  |  |  |  | - | Annoyed & Frustrated at the Tedious & Laborious Nature of Infection Control |
|  |  |  |  | - | Worries about the Others (transmission the virus to their loved ones) |
|  |  |  |  | - | Vicious Cycle of Media Consumption & Distress |
|  |  | - | Disrupted Social Support | - | Depleted/Deprived of Family Support |
|  |  |  |  |  | Closure of Elderly Centers & Churches |
|  |  |  |  |  | Intensified Pre-Existing Comorbidities |
|  | Coping | Coping with the Covid-19 Pandemic | - | Social Support through Online Technologies | - |
|  |  |  |  | Supporting by Younger People in the Neighborhood | - |

| Qing Yang  China  (Wuhan)  (15) | Emotional | - | Challenges Posed by Covid-19 | - | Inconvenience in Daily Life |
| --- | --- | --- | --- | --- | --- |
|  |  |  |  | - | Negative Emotions (Anxiety, Depression, Panic, etc.) & Psychogenic Diseases |
|  |  |  |  | - | Tight Medical Resources |
|  | Coping | Supports | - | Social Support | - |
|  |  |  |  | Technical Support | - |
|  |  | Resilience | - | Coping in Daily Life | - |
|  |  |  |  | Transcendence | - |
|  |  | Impact After the Covid-19 | Impact After the Covid-19 | - | Mental Burden (Afraid of Infection) |
|  |  |  |  | Sense of Benefit from lockdown (Better air quality) |  |
| Miriam  Verhage  Netherlands  (Leiden)  (60) | Emotional | - | Meaning in Life | - | Loss of purpose in Life |
|  |  |  |  | - | Depression & Anxiety |
|  |  |  |  | - | Loss of Autonomy & Freedom in daily life |
|  |  |  |  | - | Lack of Real & Valuable Activities |
|  |  |  |  | - | Loss of People Attention (as vulnerable persons) |
|  | Coping | Coping with the Covid-19 Pandemic | - | Self-Enhancing Comparison | - |
|  |  |  |  | Gaining Control by Following Measures | - |
|  |  |  |  | Distraction from the problem & Focused on Other Things to Keep Time Moving | - |
|  |  |  |  | Acceptance Temporary | - |
|  |  |  |  | - | Interpreting Individual “Vulnerability” |
| Eneritz Jiménez-Etxebarria  Northern Spain  (62) | Emotional | Lifestyle Changes | Lifestyle Changes | Adaptive Behavior | - |
|  |  |  |  | - | Limitation in Activities & Routine |
|  |  | Health Challenges | Health Challenges | Positive Emotion on their Health | Negative or Mixed Emotion on their Health |
|  |  |  |  | - | Physical Confinement |
|  |  |  |  | - | Psychological Confinement |
|  |  |  |  | - | Uncertainty on their Health |
|  |  | Social Challenges | Social Challenges | Use of Technology | - |
|  |  |  |  | - | Having No/Less Contacts |
|  | Coping | Coping with the Covid-19 Pandemic | - | Attitude | - |
|  |  |  |  | Reflection on the Future | - |
| Joanne Brooke  UK & the Republic of Ireland  (44) | Emotional | Protective Measures | - | Hand Wash | - |
|  |  |  |  | Social Distancing | - |
|  |  |  |  | Disinfecting Practices | - |
|  |  |  |  | Face Masks | Face Masks |
|  | Coping | Current & Future Coping Plans | - | Social Media (Positively Keep in Touch) | - |
|  |  |  |  | Weather & the Garden | - |
|  |  |  |  | Tasks to Complete | - |
|  |  | Acceptance of a Good Life  (Still a Life to Live) | - | Blessed, Lucky & Fortunate | - |
|  |  |  |  | Life still to be Live | - |
| A. R. McKinlay  UK  (45) | Emotional | - | Potential Threat to Wellbeing | - | Concern about end of life/mortality/frailty |
|  |  |  |  | - | Loss of Normality (The World Upsetting) |
|  |  |  |  | - | Healthcare Concerns (Fear of Hospitalization, Fear of leaving home infected) |
|  |  |  |  | - | Loss of Leisure/ Lack of Routine |
|  | Coping | Protective Activities & Behaviors | - | Slowing the Pace of Life (New Hobbies, More Exercise, etc.) | - |
|  |  |  |  | Benefits of Routine & Social Responsibility  (Feeling “Needed” & helping others) | - |
|  |  |  |  | Social Interaction & Support  (Reciprocal Offers of Support, Connecting) | - |
|  |  |  |  | Utilizing Skills, Experiences & Resources | - |
| Henry  Bundy  North Carolina  USA  (37) | Emotional | Loneliness Condition | - | The social isolation of COVID-19 did not exacerbate the loneliness of the already-isolated interviewees. | - |
|  |  | Loneliness, Protective, and Responsible | - | Framed their loneliness during COVID-19 in terms of necessity and responsibility, rather than pathology and shame | - |
|  | Coping | Managing Loneliness and Enduring Social Isolation | - | Manage and endure the exigencies of the COVID-19 with longstanding arrangements | - |
|  |  | Changing the Approach | - | - | The Anxieties of COVID-19 |
| Akira Teramura  Japan  Kyoto City  (67) | Emotional | - | Decrease in the Frequency of Going Out | - | Fear of Infection |
|  |  |  |  | - | Closure of Activity Places |
|  |  | - | Minimal Human Connection | - | Estranged from Family |
|  |  |  |  | - | Estranged from Residents and Friends |
|  |  | - | Decrease in Activities | - | Decreases in Amount of Conversation |
|  |  |  |  | - | Difficulty in Continuing Exercise |
|  |  |  |  | - | Reduced Leisure Activities |
|  |  |  |  | - | Changes in Roles |
|  |  | - | Disruption of Daily Life | - | Poor Eating Habits |
|  |  |  |  | - | Disturbed Sleep |
|  |  |  |  | - | Indifference toward Appearance |
|  |  | - | Deteriorating Health | - | Decline in Physical & Mental Functioning |
|  |  |  |  | - | Decrease in Motivation |
|  |  |  |  | - | Worsening of Chronic Illnesses |
|  |  | - | Increased Anxiety about the Future | - | Severe Illness due to Infection |
|  |  |  |  | - | Fear of Needing Long-Term Care |

| Pedro  Pisula  Argentina  Buenos Aires  (50) | Emotional | Network Configurations | - | The Family Network |  |
| --- | --- | --- | --- | --- | --- |
|  |  | - | Distress and Fear | The Network of Friends, Neighbors, and Other Peers | Messages from Media |
|  |  |  |  |  | Avoiding any Contact with the Outside |
|  |  |  |  |  | Transforming their Homes into an "Antiseptic Bubble" |
|  |  | - | Irritability, Anger, Boredom, and Tiredness | Perceptions of Government Policies and Decisions | The Impossibility of Seeing their Grandchildren |
|  |  | - | Sleep Disturbance | - | Nightmares, Insomnia, Changes in Waking Hours, or even Staying in Bed Longer |
|  |  |  |  | - | Increase in the Consumption of Anxiolytic Drugs without prior consultation |
|  |  | - | Perceptions and Reflections on the Future | - | Loss of Time from the Confinement in their homes |
|  |  |  |  |  | Reflections about Death |
|  |  |  |  |  | A "More Robotic" Future |
|  | Coping | Coping Resources & Strategies | - | Changing Lifestyle | - |
|  |  |  |  | Autonomy as Independent People Strengthened | - |
|  |  |  |  | Learn New Hobbies through video tutorials | - |
| Candela Agustina Loza  Argentina  Buenos Aires  (51) | Emotional & Equity in Health | Access to Regularly Scheduled Consultations | Access to Regularly Scheduled Consultations | Consultations Remotely with a Doctor | Risk Factors for the Possibility of Contagion |
|  |  | - | Control and Follow-up of the most Vulnerable Individuals with Mental Disorders | - | For Those with Preexisting Psychiatric Conditions |
|  |  |  |  |  | Preexisting Psychiatric Condition with Weak Family Support Network |
|  |  |  |  |  | Self-Harm & Suicide |
|  |  | - | Access to Chronic Medication | Technology Acted as Facilitators | Uncertainty about the Duration of Condition |
|  |  |  |  | Family Support to Get the Medications | - |
|  |  |  |  | Have Closer Relationship with a Physician | - |
|  |  |  |  | Decrease Out of Pocket in Health-Expenditures by Electronic Prescriptions |  |
|  |  | Acute & Emergent Consultations | Acute & Emergent Consultations | Use of the Emergency Medical Care System of the Government | The Consultation Was Resolved by  Self-Medication |
|  |  | - | Fear and Misinformation | - | Possibility of Contagion |
|  | Coping | Information & Communication Technologies | Information & Communication Technologies | Easy Access to the Health Services | Lack of Skills in the Management of New Technologies |
|  |  |  |  | - | Physical Examination as a Crucial Tool for Diagnosis |

| Heather R.  Fuller  USA  North Dakota  & Minnesota  (38) | Emotional | - | Increased Feeling of Loneliness | - | Lack of Control |
| --- | --- | --- | --- | --- | --- |
|  |  |  |  | - | Feeling of Loss |
|  |  | Decreased Feeling of Loneliness | - | Accustomed to Being Alone | - |
|  |  |  |  | Staying connected using phones/technology | - |
|  | Coping | Coping Strategy | - | Staying connected using phones/technology | - |
| Kulmala J  Finland  (52) | Emotional | - | Reduction in The Size of the Personal Network | - | Avoiding all places with a lot of people |
|  |  |  |  | - | Fear (own or others) of the virus |
|  |  |  |  | - | Restricting contacts even with the closest family |
|  |  |  |  | - | Hobbies has been closed |
|  |  |  |  | - | Use of digital tools were perceived as difficult and were not applied |
|  |  |  |  | - | Relatives prohibited contacting other people |
|  |  |  |  | - | A relative/friend is a caregiver for someone else and cannot leave home |
|  |  |  |  | - | Meetings outside impossible due to own of other person’s sickness or disability |
|  | Coping | The Pandemic Had No Influence on Personal Networks at all | - | Phone contacts with relatives and friends were as common as previously | - |
|  |  |  |  | Friends and family visited regardless of restrictions or children live close or at the same house | - |
|  |  |  |  | Current personal social network was seen as fulfilling | - |
|  |  |  |  | Enjoying time alone and having no obligations to leave home |  |
|  |  | Modifications or Increase in Contacting Other People | - | Increased Phone Contacts | - |
|  |  |  |  | Spending more time with partner | - |
|  |  |  |  | Increase in Importance of Pets | - |
|  |  |  |  | Hobbies, i.e., physical activity groups, organized online | - |
|  |  |  |  | Video, internet and WhatsApp contacts with the family started | - |
|  |  |  |  | Relatives, friends and neighbors were met outside and with safety distances | - |
|  |  |  |  | Applying safer ways of greeting and meeting people, i.e., not shaking hands anymore, using face masks | - |

| Tiilikainen E  Finland  (53) | Emotional | Social Contacts | - | Frequently Contacts with family and friends | - |
| --- | --- | --- | --- | --- | --- |
|  |  |  |  | Increase Using of WhatsApp and video calls |  |
|  |  | Daily Chores and Activities | - | housekeeping | - |
|  |  |  |  | Reading, knitting and doing crosswords | - |
|  |  |  |  | Going for walks with their Pets. | - |
|  | Coping | Places and Seasonal Changes | - | Going outdoors and enjoying nature | - |
| Siu‑Ming Chan  Hong Kong  (55) | Emotional | life Philosophy | - | A Sense of Altruism towards the Young Generation | - |
|  |  |  |  | Not Afraid of Death | - |
|  |  | Economic Security | - | Retired and Receiving Social Welfare from the Government | - |
|  |  | Telecommunication | - | Social Interaction of Elders be Compensated | - |
|  |  | - | Role of Community Organizations & Social Workers | - | Closure of Elder Community Centers |
|  |  |  |  | - | Being Hopeless and Disappointed |
|  | Coping | Positive Coping Strategies | - | Sports (‘Tai-chi’ and ‘Qi-Qong’) | - |
|  |  |  |  | Entertainment | - |
|  |  |  |  | Playing Music | - |
| Pranab Mahapatra  India  (48) | Emotional | - | Risk Appraisal & Feeling Vulnerable | - | Being aware of their Frailness, heightened Susceptibility to the COVID-19, and the consequential Severity |
|  |  |  |  | - | Fear of Losing Own or Partner’s Life |
|  |  | Safeguarding against  COVID-19 | - | Adherence to COVID-19 Prevention Measures | - |
|  |  |  |  | Social Distancing & Limiting Outsider Exposure | - |
|  |  |  |  | Adopting Health-Promoting Behaviors | - |
|  | Coping | Managing Routine Health Care & Emergency | Managing Routine Health Care & Emergency | Continuing Routine Health Care | - |
|  |  |  |  | - | Fear of Visiting Hospital vs Managing Care |
|  |  |  |  | Managing Emergency by their Physicians | - |
|  |  |  |  | Harnessing Available Resources & Support Network | - |
|  |  | Pursuing Mental & Psychological Well-Being | Pursuing Mental & Psychological Well-Being | - | Perennial Invisible Stress |
|  |  |  |  | Rejuvenating Spousal Relationship | - |
|  |  |  |  | Connectedness with Family & Friends | - |
|  |  |  |  | Spiritual Well-Being | - |
|  |  |  |  | Rediscovering their-selves | - |

| Rasoul Norouzi Seyed Hosseini  Iran  (56) | Emotional | The experience of Increasing Psychosocial Isolation & Mortality | The experience of Increasing Psychosocial Isolation & Mortality | | Ignoring & Increasing the Restrictions on Sports & Exercise!!! | - |
| --- | --- | --- | --- | --- | --- | --- |
|  |  |  |  |  | - | Being Ignored by the Authorities in Plans |
|  |  |  |  |  | - | Feeling Psychological Stress & Isolation |
|  |  |  |  |  | - | Depression & Reduction of Opportunities |
|  |  | - | The Marginalization of Old Age Sports | | - | authorities neglect senior sports and overlook the health needs and requirements for older athletes in their reopening policies |
|  |  | - | Unwanted Changes in Lifestyle & Mood Disorders | | - | developed obsessive-compulsive disorder, mood swings, insomnia, and dietary changes |
|  |  |  |  |  | - | weakness and physical problems |
|  |  |  |  |  | - | disruption of the lifestyle and the routine |
|  | Coping | Active & Creative Sports Activism | - | | Design their Exercises in their Quarantine Environments | - |
| Mohammad  Asgari  Iran  (57) | Emotional | - | Empty Nest Experience | | - | Feeling Lonely |
|  |  |  |  |  | - | Isolation & Reclusion |
|  |  |  |  |  | - | Feeling Dreary |
|  |  | - | Hypersensitivity | | Concerns about the health of family members | Concerns about the health of family members |
|  |  |  |  |  | Fear of getting sick due to going to clinics | Fear of getting sick due to going to clinics |
|  |  |  |  |  | - | Concerns about skin problems caused by the use of disinfectants |
|  |  |  |  |  | Weight gain and related concerns | Weight gain and related concerns |
|  |  |  |  |  | Financial Concerns | Financial Concerns |
|  |  | - | Psychological Distress | | - | Fear & Anxiety |
|  |  |  |  |  | - | Feeling Hated |
|  |  |  |  |  | - | Gloomy Mood |
|  |  |  |  |  | - | Obsession |
|  |  |  |  |  | - | Boredom & Confusion |
|  |  |  |  |  | - | Calmness Deprivation |
|  |  |  |  |  | - | Aggression |
|  |  | - | Personal Problems | | - | Waiting to Be Illness |
|  |  |  |  |  | - | Loss of Autonomy & Freedom in daily life |
|  |  |  |  |  | - | Perception of Disease Attribution to the Elderly |
|  |  |  |  |  | - | Unpredictability of Disease |
|  |  |  |  |  | - | Loss of Leisure Time |
|  |  |  |  |  | - | Feeling Regret |
|  |  | - | Interpersonal Problems | | - | Family Perturbation |
|  |  |  |  |  | - | Receiving Caveat from Others |
|  |  | - | Tension Resulted from Conditions | | - | Upset about not holding ceremonies and rituals |
|  |  |  |  |  | Compatibility problems with changes | Compatibility problems with changes |
|  |  |  |  |  | - | Discomfort from the mass media |
|  | Coping | Not Mentioned | Not Mentioned | | - | - |
| Jonaid M. Sadang  Philippines  (69) | Emotional | - | Everyday Struggles | | - | Prohibition of going outside and limitation of activities |
|  |  |  |  |  | - | Insufficient government support |
|  |  |  |  |  | - | Lack of sources of income or livelihood |
|  | Coping | Embracing Reality | Embracing Reality | | Acceptance | - |
|  |  |  |  |  |  | Left with no other choices |
|  |  | Finding Things to Enjoy | - | | Practice of religious rituals and prayers | Practice of religious rituals and prayers |
|  |  |  |  |  | Alternative diversional activities to alleviate boredom | - |
|  |  | Health Consciousness | - | | Observing and taking precautions regarding one’s health | - |
|  |  |  |  |  | - | Misconceptions regarding COVID-19 |
|  |  | Cultivating a Strong Spirituality | - | | Belief in one’s fate | Belief in one’s fate |
|  |  |  |  |  | Surrendering self to God and being left with no fear | Surrendering self to God and being left with no fear |
|  |  |  |  |  | Strong belief and trust to God | Strong belief and trust to God |
|  |  |  |  |  | Considering the situation as a blessing from God | Considering the situation as a blessing from God |
| Ilaria Falvo  Italy  (58) | Emotional | - | Impact on the individual level | Between fear of going out and a feeling of reclusion | - | Between the risk of being intubated or dying |
|  |  |  |  |  | - | Depression & Loneliness |
|  |  | - | Impact on the micro-social level | The dual role of the other | - | No respecting on COVID-19 prevention measures by strangers |
|  |  |  |  |  | - | No respecting on COVID-19 prevention measures by their Peers |
|  |  |  |  |  |  | No respecting on COVID-19 prevention measures by the younger people |
|  |  | - | Impact on the meso-social level | Between protection and stigmatization | - | Feeling Stigmatized & Discriminated |
|  |  |  |  |  | - | Two opposite representations of the elders:  As Vulnerable Group  As Leper Group |
|  | Coping | - | Impact on the macro-social level | Gestation of a new world | Deepen our own meaning of life | - |
|  |  |  |  |  | People will be wiser | - |
|  |  |  |  |  | People will be more grateful for what they have | - |

| Sabrina Cipolletta  Italy  (59) | Emotional | - | Social Networks | - | Distance from relatives |
| --- | --- | --- | --- | --- | --- |
|  |  |  |  | - | Distance from friends and acquaintances |
|  |  |  |  | - | Previous withdrawal/ Neighbors’ withdrawal |
|  |  |  |  | - | Cohabiting partner |
|  |  |  |  | - | Physical closeness of children |
|  |  |  |  | - | Occasional family visits |
|  |  |  |  | - | Relationship with neighbors |
|  |  |  |  | - | No relationships maintained |
|  |  | Changes in daily life | Changes in daily life | Physiological and psychological changes | Physiological and psychological changes |
|  |  |  |  | - | Isolation as imposition |
|  |  |  |  | Isolation as responsibility | Isolation as responsibility |
|  |  |  |  | Changes of habits/ Changes as opportunity | Changes of habits/ Changes as opportunity |
|  |  |  |  | Changes in a spiritual sphere | - |
|  |  |  |  | Reorganization of activities | - |
|  |  |  |  | Cessation of help in housework | Cessation of help in housework |
|  |  |  |  | Loss of grandparents’ role | - |
|  |  |  |  | - | Limitations to active ageing |
|  |  | Emotions Concern | Emotions Concern | - | Fear & Anger |
|  |  |  |  | - | Incredulity & Obsession |
|  |  |  |  | - | Sadness & Loneliness |
|  |  |  |  | Peacefulness | - |
|  |  | Exploited resources and strategies | Exploited resources and strategies | External resources | External resources |
|  |  |  |  | Internal resources: practical and emotional issues | Internal resources: practical and emotional issues |
|  |  |  |  | Internal resources: rediscovery of hobbies | Internal resources: rediscovery of hobbies |
|  |  |  |  | - | To go out and skip the anti-contagion norms |
|  |  |  |  |  |  |
|  |  | Use of Media | -(15) (13 (17) To go out and skip the anti-contagion norms (4) Attempt to find the origin of the virus | Telephone contacts with relatives/ with friends/ with neighbors/ with colleagues | - |
|  |  |  |  | - | Technological skills & Difficulty of using technologies |
|  |  |  |  | Efficient means | Inefficient means |
|  |  |  |  | Proactivity in learning to use new media | - |
|  |  |  |  | - | Physical limits in using technologies |
|  |  |  |  | - | Lack of learning |
|  |  |  |  | Personal preferences | Personal preferences |
|  |  |  |  | Television & Infodemic | Television & Infodemic |
|  | Coping | View of the Future | - | Hope | - |
|  |  |  |  | Long-term duration | Long-term duration |
|  |  |  |  | Long-term consequences | Long-term consequences |

| Trish  Hafford-Letchfield  UK  (46) | Emotional | - | Risk factors for LGBT+ older people and organizations | - | Risk factors experienced by LGBT+ older people  (Loss of physical and social contact/ Fragility of basic needs/ Reduced family support, and etc.) |
| --- | --- | --- | --- | --- | --- |
|  |  |  |  | - | Specific risks for trans people  (Increased opportunities for harassment/ Increase in anti-trans environment, and etc.) |
|  |  |  |  | - | Risk factors for LGBT+ organizations  (Lack of information in health and social care about LGBT+ community / Increased fragmentation of services, and etc.) |
|  |  | Care practices in LGBT+ lives | Care practices in LGBT+ lives | Secure relationships/partnerships | - |
|  |  |  |  | Offering accommodation to partners | - |
|  |  |  |  | Increased visibility of concealed relationships | - |
|  |  |  |  | Active outreach to family/friends | - |
|  |  |  |  | Effect of rurality on networks | - |
|  |  |  |  | Reconnecting/repairing relationships | - |
|  |  |  |  | - | Fear of formal care |
|  |  |  |  | Increase in volunteers | - |
|  |  |  |  | Advocacy in transfer to formal care | - |
|  |  | Strengths and benefits of networking | - | Opportunities to connect with neighbors | - |
|  |  |  |  | Kinder communities | - |
|  |  |  |  | Being aware of other's needs | - |
|  |  |  |  | Increase in volunteers | - |
|  |  |  |  | Increase take up of services online | - |
|  |  |  |  | Role of anonymity | - |
|  | Coping | Learning from communication and provision in a virtual world | - | Improved virtual service for trans | - |
|  |  |  |  | New peer networks | - |
|  |  |  |  | Increase in volunteers | - |
|  |  |  |  | Increase take up of services online | - |
|  |  |  |  | Stretched resources | - |
|  |  |  |  | Costs and benefits of adapting services to virtual delivery | - |
|  |  |  |  | New peer networks | - |

| Evi M. Kremers  Netherlands  (61) | Emotional | Social behavior during the COVID-19 outbreak | Social behavior during the COVID-19 outbreak | Maintenance of contact | - |
| --- | --- | --- | --- | --- | --- |
|  |  |  |  | Adaptation (Better days will come!) | - |
|  |  |  |  | - | Less contact |
|  |  | Emotional behavior during the COVID-19 outbreak | Emotional behavior during the COVID-19 outbreak | Perception during COVID-19 outbreak | Perception during COVID-19 outbreak |
|  | Coping | Motivation to expand social network due to COVID-19 | - | Motivation & Expanding the network | - |
| Carmen Llorente-Barroso  Spain  (63) | Emotional | - | Negative Effects of the Pandemic on the Well-Being of the Elderly Aged over 60 | - | Emotional impact of the lack of physical contact with their loved ones |
|  |  |  |  | - | Perception of loneliness during the confinement |
|  |  |  |  | - | Negative perception about the passing of time |
|  |  |  |  | - | Concern and anxiety triggered by uncertainty (uncertain situation & fear of sickness) |
|  |  |  |  | - | Sadness at the loss of loved ones |
|  | Coping | Emotional, humanizing and operational role of ICT (Information and Communication Technology) in enhancing well-being and mitigating fear of the elderly during the pandemic | - | Emotional support of ICT for the contact with their loved ones | - |
|  |  |  |  | Operational role of ICT in mitigating fear through the avoidance of infection | - |
|  |  |  |  | ICT as entertainment and distraction tools during the confinement | - |
|  |  | Learning ICT and ICT-enhanced learning as motivational triggers of effort and personal autonomy of the elderly | - | Learning ICT and self-satisfaction | - |
|  |  |  |  | Use of ICT to foster hobbies and to address personal concerns | - |

| Jessica R Daly  USA  (39) | Emotional | - | Impact of the Quarantine on Health and Well-Being | - | Negative impact of the quarantine on participants’ physical health (weight gain, decreased mobility, worsening of existing health conditions, and/or development of new conditions due to decreased physical activity). |
| --- | --- | --- | --- | --- | --- |
|  |  |  |  | - | Negative impact of the quarantine on participants’ mental health (limited opportunity to engage with others, increased feelings of grief associated with being alone, anxiety and depression, long lasting side effects of the pandemic) |
|  | Coping | Communication Innovation and Technology Use | - | Developed to mitigate the social isolation stemming from the quarantine (web access to the church for Sunday services, and to their friends’ group) | - |
|  |  |  |  | The use of digital medicine | - |
|  |  | Effective Ways of Coping with the Quarantine | - | Having control in how perceive the quarantine as either an opportunity or a threat (supported their “neighborhoods”, celebrating uncommon holidays | - |
|  |  | Improving Access to Technology and Training | - | Finding desirable peer-educator model to teach them how to use the technology | - |
| Qianyun Wang  China  (65) | Emotional | - | Considered themselves as a vulnerable, high-risk population requiring extra care. | Using Protective measures to reduce the burden of the young ones. | Using Protective measures to reduce the burden of the young ones. |
|  |  |  |  | - | Fear of Death |
|  |  | - | Being prohibited from various social activities | - | No attend the funeral of their loved ones |
|  |  |  |  | - | Ageist policies and ageism in the media (society seemed to be “giving up on seniors”) |
|  |  | - | Experiencing racial discrimination | - | Heightened discrimination as a consequence of the pandemic and their ethnicity |
|  |  |  |  | - | Created discomfort and distress and causing further isolation |
|  |  |  |  | - | worry about anti-Asian sentiment in the U.S. |
|  | Coping | Accumulated life experiences of ageing to have resilience. | - | - | - |
|  |  | Family Roles as older parents and grandparents | - | Strong feelings of connection with their family interactional in pandemic | - |
|  |  | Use of technology | - | To access information about the pandemic and connect with their families and friends | - |
|  |  |  |  | facilitated transition to virtual social activities | - |
| Arlinde Johanna Dul  Netherlands  (16) | Emotional | - | Social Issues | - | Challenges concerning encounters with family, friends, and other people in daily life (staying at home, reduce social contacts, no physical contact outside the household) |
|  |  | - | Environmental Issues | - | Challenges concerning environmental measures which are the result of national and local COVID-19 policies in organizations, buildings, or shops (following national and local measures, no attention behavior of other people in public space) |
|  |  | - | Health Subjects | - | Challenges concerning the participants own health or the health of relatives (perceived risk for contamination and/or transmission, own stance in the situation) |
|  | Coping | Selection | Selection | - | Stop activity - forced |
|  |  |  |  | - | Reducing activity |
|  |  |  |  | Changing goals (alternative activities or extent activities) | - |
|  |  | Optimization | - | Anticipatory planning (avoid busy places/ change routes/share space or stuff/ plan at specific times) | - |
|  |  |  |  | Changing environments (make home more comfortable and functional) | - |
|  |  |  |  | Balancing the tensions (accept the situation or rationalize/evade the rules/ obey the rule/ protecting self or others/ following own standards) | - |
|  |  |  |  | Pushing self (doing activities) | - |
|  |  |  |  | Relying on previous routines | - |
|  |  | Compensation | - | Modifying (alternative places/ extra hygienic measures/ going alone/ perform activity from home/ pursue activity for a shorter period/ social distancing | |
|  |  |  |  | Receiving help | Receiving help |
|  |  |  |  | Substituting (delivery/ online or other sources of communication) | - |

| Maria Alice Cavalcante Gomes  Brazil  (64) | Emotional | Spirituality and pre-pandemic pleasurable activities | Spirituality and pre-pandemic pleasurable activities | Increase in spiritual activities and the possibility of strengthening their beliefs based on their experiences | Losing the activities related to spirituality that were part of their daily lives before the social isolation ( religious meetings, dialogues developed with neighbors, visits to friends) |
| --- | --- | --- | --- | --- | --- |
|  |  | Missing the extra-household routine and family life | Missing the extra-household routine and family life | Usefulness of phone calls as a positive coping measure | Nostalgia for leaving home, going for walks, and developing routine activities |
|  |  |  |  | - | Keep away from their relatives |
|  |  | - | The signs/symptoms experienced during the infection | - | Misconceptions about the symptoms of the disease and its similarity to the seasonal flu |
|  |  |  |  | - | Bad symptoms (rapid worsening of the condition, need for mechanical ventilation, and associated prolonged dyspnea with risk factors such as overweight) |
|  |  | - | Fear of dying | - | Made more distressed by the sadness and fear of loss of family life |
|  | Coping | The construction of a new routine | - | Developing activities such as taking care of the plants, organizing the house, cooking, talking to their spouse | - |
|  |  |  |  | Increase in communication with the spouse, which enables an improvement in interpersonal relationships | - |
|  |  | The strategies adopted for the prevention of COVID-19 | - | Complying with pandemic health rules | - |
|  |  |  | - | Feeling need for changes in routine to adapt to the prevention strategies of COVID-19 | - |
| Ahmet Kosar  Turkey  (72) | Emotional | - | No talking about the Virus |  | Consider virus as the main problem  whether coronavirus was produced by natural means or artificially |
|  |  |  |  |  | Didn't know much about the disease |
|  |  | Information Resources About Corona Virus |  | From internal and foreign medias | - |
|  |  |  |  | Scientific articles & Internet | - |
|  |  | - | Attitudes on Covid-19 | - | Daily Life routines Changes |
|  |  | - |  | - | Intubated at home |
|  |  | - |  | - | Loss of social connections |
|  |  | - | Difficulties Experienced | - | Loss of relations with people |
|  |  | - |  | - | Distress & Depression |
|  |  | - |  | - | Changes in house works |
|  |  | - |  | - | Withdrawal from the bank & internet infrastructure & bill payments |
|  |  | - |  | - | Pain due to inactivity |
|  | Coping | New information technologies | - | Video-talk technology, cooperation between relatives and neighbors | - |
|  |  | Daily Routines | - | Support of relatives and close environment | - |
|  |  | Saving Health Status | - | Sport activities at home & Communication | - |

| Sofi Fristedt  Sweden  (71) | Emotional | - | My world closed down | - | Struggled to handle the abrupt cancellations of health care (general practice visits, surgery) |
| --- | --- | --- | --- | --- | --- |
|  |  |  |  | - | Losing cherished activities (choir rehearsals, cooking groups, in/ outdoor exercises, social activities) |
|  |  | Negotiations, adaptations and prioritizations to manage staying at home | Negotiations, adaptations and prioritizations to manage staying at home | The freedom to make individual but informed decisions and negotiations around activities | Postpone health care visits with being afraid of getting the virus. |
|  |  |  |  | Remain independent (shopping online/by delivery- | Depriving from visiting their family, friends and neighbors |
|  |  | Contextual barriers and facilitators to sustain occupational participation | Contextual barriers and facilitators to sustain occupational participation | Doing routines (gardening, genealogy, playing cards or using the computer) | More challenging to live alone during the pandemic. |
|  |  |  |  | Appreciated the pets for enabling the maintenance of daily routines and social interactions. | Social contacts suddenly became reduced to a minimum |
|  |  |  |  | Increased consumption of news to stay up to date on the developments of the pandemic. | - |
|  |  | - | Considerations about my own and other’s health and wellbeing | - | Worried about becoming ill with COVID-19, especially those experiencing additional risk factors. |
|  |  |  |  | - | concerned about the reported dramatic course of the disease |
|  | Coping | Strong Personality | - | Helped them handle being alone | - |
|  |  | Low-key life is positive |  | Forgetting fatigue depression, and other previous problems | - |
| Paxton Bruce  Canada  (43) | Emotional | - | Fear and Anxiety | - | less afraid of the virus itself than the impact of the fear of the virus |
|  |  |  |  | - | highest risk for severe complications of COVID-19 |
|  |  |  |  | - | removed the hope of returning to a normal life |
|  |  | - | Feelings of Sadness and a Sense of Loss | - | change in daily routines (physical distancing measures and social restrictions) |
|  |  | - |  |  | increased isolation |
|  |  | - |  |  | changes in personal and professional purpose and roles |
|  | Coping | Negotiating Social Interaction | - | Carefully weigh the risks associated with in-person interactions | - |
|  |  |  |  | New ways of interacting | - |
|  |  |  |  | (Dis)Connecting through technology. | - |
|  |  | Growth Through the COVID-19 Pandemic | - | Developing new activities | - |
|  |  |  |  | Dwelling more deeply in life | - |
|  |  |  |  | Shared suffering | - |
| Moumita Das  India  (49) | Emotional | Social connectedness and paradoxes: emic view | Social connectedness and paradoxes: emic view | A paradox in elderly social relationships that are encapsulated (Friends vs Family)!! | A paradox in their social relationships that are encapsulated (Friends vs Family)!! |
|  |  |  |  | - | The supportive joint family structure disrupted with nuclearize leading to an increase in stress levels for them |
|  |  |  |  | - | Detachment from proximal members caused mental distress, loneliness and depression |
|  |  |  |  |  | Feeling distressed for being away from charitable activities |
|  |  | Interpreting the ‘pandemic anxieties’ in elderly social lives: etic view | Interpreting the ‘pandemic anxieties’ in elderly social lives: etic view | Increase interaction with neighbors and friends instead of family and relatives | An ongoing paradox in a relationship, it has disturbed the elderly life culturally |
|  |  |  |  | - | Anxious about their adult children’s job securities |
|  | Coping | Coping strategies in this pandemic times: emic view | - | Involving themselves in certain activities confined in their home (reading books, involving in spiritual activities, cooking food, watching television, doing free-hand exercises, learning e-technologies, donate food and grants-in-aid) | - |
|  |  |  | - | Involving in regular sanitization and hand-washing regimes to avert virus infection | - |
|  |  |  | - | Give priority in society to allow their guiding edge to control the younger generation | - |
|  |  | Preparedness for the future: emic view | - | Learned to manage the losses incurred during the lockdown period to regain psychological and physical well-being | - |
| Prince Chiagozie Ekoh  Nigeria  (68) | Emotional | - | Diminishing Material Support | - | The economic hardship (dependency on their children & social distancing reduced it) |
|  |  |  |  | - | Lockdown and movement restrictions impacted on the livelihood of people |
|  |  | - | Diminishing Intangible Support | - | Limited social contact between them and their children (they live in cities) |
|  |  |  |  |  | Children were encouraged to stay away from the elderly to keep them safe (vulnerability) |
|  |  |  |  | - | Deprivation many older people of in-person communication |
|  | Coping | - | Unequal access to government’s relief materials | - | Unequal distribution in rural areas |
|  |  |  |  | - | Distribution to the entire rural community with no special considerations for elderly |
|  |  | - | Inadequate nutritional and medical needs | - | Elderly had no access to food |
|  |  |  |  | - | Elderly had no access to medical needs |
| Delali  Adjoa Dovie  Ghana  (66) | Emotional | Socio-cultural dimension | Socio-cultural dimension | Closing of schools made grand-children available for the elderly | Vulnerable connotation between them and their grandchildren |
|  |  |  |  | Turn to the use of the internet by some grandchildren | Withdrawal from responsibility |
|  |  | - | Health dimension | - | Fear of visiting their physicians |
|  |  |  |  | - | Losing control on their chronic diseases |
|  |  | Humanitarian dimension | - | Be benefited from donations from all walks of life | - |
|  |  | - | Emerging issues | - | Feeling loneliness |
|  |  |  | Emotional dimension | - | Loneliness, immobility to socialize, tiredness beyond the ordinary level, inability to socialize among others |
|  | Coping | Social coping | - | Interaction through virtual space (WhatsApp, Facebook, Twitter, etc.) or telephone | - |
|  |  |  |  | Sleep or more relaxation, listening to news on both television and radio | - |
|  |  | Reactive coping | - | Intervention oriented activities (taking care of the neighbors children) | - |
|  |  |  |  | The problem-focused coping mechanisms | - |
|  |  |  |  | The emotion-focused coping mechanisms | - |

| Kathleen Melei  USA  (73) | Emotional | Valued Activities | Valued Activities | Participated in exercise classes online | Losing exercise, walking, leisure, eating out with friends, cleaning, baking, quality time with family | |
| --- | --- | --- | --- | --- | --- | --- |
|  |  |  |  | - | Losing spontaneous interaction and a physical experience | |
|  |  |  |  | - | Missed hugging | |
|  |  | - | Concern about Others and Self | - | More concern for family, friends, and society rather than concern for self; concern for the younger generation | |
|  |  | - |  | - | Concerns with access to healthcare | |
|  |  | - |  | - | Feelings of uncertainty, frustration, and sadness with society | |
|  |  | - |  | - | The most difficult part of the pandemic: the lost “sense of being free,” comparing quarantine to a prison sentence | |
|  |  | - | Perceptions of Aging | - | Death and its impact on their relationships | |
|  |  | - |  | - | The pandemic “robbed” them of a year | |
|  |  | Sustained Social Participation | Sustained Social Participation | Utilizing Zoom and Snapchat, Facebook and phone calls or spending time individually with grandchildren rather than seeing them all at once | Changes in their ability to participate in desired occupations (narrow their social circles, limiting social connections) | |
|  |  | Disrupted Social Participation | Disrupted Social Participation | Could still interact virtually (missed hugging their children and grandchildren) | Could not visit family or complete activities in other states | |
|  |  | - | - | - | Decrease in physical activity due to the restrictions, and, especially during winter, | |
|  | Coping | Coping Strategies | - | Exercise or home management to cope with changes in activity | - | |
|  |  |  |  | Utilized yoga, meditation, and environmental adaptations | - | |
|  |  |  |  |  | - | |
|  |  | - | Socio-political Context | - | Decreased participation in activities and disrupted quality of relationships | |
|  |  |  |  | - | Feeling disillusioned and saddened by close family and friends’ behavior, beliefs in relation to the pandemic and the election | |
|  |  |  |  |  | Feelings of frustration and disappointment with the behavior and beliefs of the community at large | |
|  |  |  |  | - | Large-scale societal concerns regarding death, social security, government transparency, and the media. | |
| Salina Jivan  USA  (74) | Emotional | Impact | Impact | Facing A New Reality | | Lost Time and Freedom |
|  |  |  |  | - | | Loss of Meaningful Social Participation |
|  | Coping | Adaptations | - | Staying Connected | - | |
|  |  |  |  | Remaining Engaged in Purposeful Occupations | - | |
|  |  |  |  | Silver Lining (learned to value family and friends & the value of connection) | - | |
|  |  | Future Outlook | - | New Normal | - | |
|  |  |  |  | Cautious Community Return | - | |
|  |  |  |  | Looking Forward to Social Gatherings | - | |
